# Development and validation of the Workplace Learning Inventory in Health Sciences Education: a multimethod study

**DOI:** 10.1007/s10459-023-10295-y

**Published:** 2023-11-08

**Authors:** Evelyn Steinberg, Stephan Marsch, Takuya Yanagida, Laura Dörrenbächer-Ulrich, Christopher Pfeiffer, Petra Bührle, Lukas Schwarz, Ulrike Auer, Christin Kleinsorgen, Franziska Perels

**Affiliations:** 1 Vice-Rectorate for Study Affairs and Clinical Veterinary Medicine, University of Veterinary Medicine Vienna, Vienna, Austria; 2 Department of Educational Science, Saarland University, Saarbrücken, Germany; 3 Clinic for Swine, University of Veterinary Medicine Vienna, Vienna, Austria; 4 University Hospital for Small Animals, University of Veterinary Medicine Vienna, Vienna, Austria; 5 Centre for E-Learning, Didactics and Educational Research, University of Veterinary Medicine, Hannover, Germany

**Keywords:** Academic emotion, Academic motivation, Health sciences education, Learning environment, Learning strategies, Self-regulated learning, Questionnaire, Workplace learning

## Abstract

Health sciences students face many challenges in regard to clinical practical learning. A better understanding of student learning is required to address student needs in this crucial phase. The theory of self-regulated learning provides a comprehensive view of learning and could serve as a basis for further research. There are instruments to assess self-regulated learning in preclinical academic learning. However, there are no such instruments for workplace learning. The aim of the present study is to provide a comprehensive inventory from which researchers can select those scales that are relevant to their research questions in the investigation of workplace learning. Hence, the aim is to develop and validate a set of scales to assess undergraduates’ workplace learning in health sciences education in four areas (cognition, motivation, emotion, and context) on two levels (the learning process level and the metalevel). Study 1 is a qualitative multimethod study to identify indicators and develop items. It integrates the perspectives of students, teachers, and researchers and includes six steps: literature review, interviews, synthesis, item development, expert review, and cognitive pretesting. This study yields a set of scales for each area on both levels. Study 2 is a quantitative study to assess the psychometric properties. The results show acceptable values in terms of unidimensionality, reliability and validity for each of the 31 scales. The newly developed Workplace Learning Inventory is comprehensive; the scales are relevant to workplace learning and short enough that their administration is feasible in the workplace setting. The rigorous process of questionnaire development contributes to the validity of scales. By providing the Workplace Learning Inventory, we hope to encourage research on workplace learning in health sciences education from an educational psychology perspective.

**Supplementary Information:**

The online version contains supplementary material available at 10.1007/s10459-023-10295-y.

## Introduction

Many students struggle in transitioning from preclinical academic learning to clinical practical learning in health sciences education, and some continue to struggle even after they have become familiar with the workplace environment (Atherley et al., 2019; Godefrooij et al., 2010; Teo et al., 2011; Westerman & Teunissen, 2013; White, 2007). To better address students’ needs in this crucial phase, a clearer understanding of different aspects of student learning is needed. A comprehensive understanding of learning is a key point since learning should not only result in high achievement. Additionally, student motivation and well-being are considered important (Dai & Sternberg, 2004; Fares et al., 2016; Frajerman et al., 2019).

Research on learning in health sciences education is seen as interdisciplinary but is predominantly informed by the health research domain (Albert et al., 2020). Health sciences education research should be informed by other disciplines, one of which is psychology and, more specifically, educational psychology. Educational psychology research has resulted in a comprehensive understanding of learning, known as self-regulated learning (SRL) (Schunk & Greene, 2018). SRL includes multiple components, such as cognition, motivation, emotion, and the perception of the learning environment as well as the metalevel of learning, considered in terms of metacognition and regulation of motivation and emotion (Ben-Eliyahu, 2019; Ben-Eliyahu & Bernacki, 2015; Panadero, 2017; Pintrich, 2004; Wolters, 2003).

In the last decade, this comprehensive view of learning has been adopted in health sciences education (Artino et al., 2015; Cleary et al., 2013; Hayat et al., 2020; van Houten-Schat et al., 2018), and there are several studies on SRL in medical education. While in educational psychology research SRL is viewed as a multifaceted construct (Pintrich, 2004) and with differentiated underlying mechanisms (Panadero, 2017), health sciences education research on SRL seems not to have adopted this differentiated view (van Houten-Schat et al., 2018). Recognizing the multifaceted nature of SRL could help to understand the underlying mechanisms of student learning in health sciences education. Furthermore, existing studies in health sciences education research mainly address learning in the preclinical academic setting and are often based on qualitative or cross-sectional quantitative methods (van Houten-Schat et al., 2018). However, not only learning in the preclinical academic setting (abbreviated as academic learning; e.g., Biwer et al., 2023) but also undergraduate learning in the practical clinical setting (abbreviated as workplace learning; e.g., Sagasser et al., 2017) is of great interest, including the transition from academic learning to workplace learning (Westerman & Teunissen, 2013).

Academic learning focuses on individuals learning of theoretical foundations in a learning environment that students can create to a large extend by themselves according to their needs. It also focuses on individuals learning of specific motor skills or social skills in a highly structured environment provided by a teacher within the framework of a propaedeutic course. In contrast, workplace learning focuses on individuals learning in a complex learning environment. The workplace can be a clinic, a clinical practice or a company. In line with self-determination theory (Deci & Ryan, 2012), students require supportive conditions for psychological growth. Hence, in such settings undergraduates do not only need to experience and attain competency but also to develop role autonomy, join the community of practice and interact with patients (Cruess et al., 2018; Morris & Behrens, 2013b).

From an educational psychology perspective, there are only a few studies about workplace learning in health sciences education, and there is a lack of recognition of the multifaceted nature of SRL as well as a lack of quantitative multivariate and prospective longitudinal studies of workplace learning in health sciences education (van Houten-Schat et al., 2018).

A prerequisite for such studies is the availability of appropriate instruments for assessing SRL in health sciences education, such as questionnaires. Established questionnaires on learning in higher education often (1) focus on academic learning (e.g. Pintrich et al., 1993), (2) address single components of learning and/or (3) are characterized by long scales (Duffy et al., 2018; Strand et al., 2013; Wolters & Benzon, 2013). To analyze and assess workplace learning, instruments are needed that (1) focus on workplace learning, (2) recognize the multifaceted nature of SRL and address the multiple components and aspects of learning and (3) provide different short scales to be feasible in longitudinal studies. The aim of the present study is to provide a comprehensive inventory from which researchers can select those scales that are relevant to their research questions in the investigation of underlying mechanisms in workplace learning.

### A component-based conceptual framework for workplace learning

Students face cognitive, motivational, and emotional challenges when transferring from academic to workplace learning. Educational psychology research provides different theoretical frameworks for integrating such different components of learning (Dai & Sternberg, 2004; Slavin, 2018). We refer to the theory of SRL because of its broad view of learning and its relevance for academic success while considering motivation and affect. According to Pintrich (2004), the ideal self-regulated learner sets goals and is able to regulate cognition, motivation/affect, behavior and context to achieve a goal. SRL models can be divided into more component-based models, such as Pintrich's conceptual framework for assessing motivation and SRL (Pintrich, 2004) or Boekaerts’s six component model of SRL (Boekaerts, 1996), and more process-based models, such as Zimmerman’s cyclical phases model (Zimmerman, 2008). Because of the comprehensive view of workplace learning and because component-based models emphasize the diversity of aspects that are relevant to learning, the foundation of our study is a component-based conceptual framework for assessing workplace learning drawing on Pintrich’s differentiation between areas of regulation and Boekaerts’s differentiation of levels.

Following Pintrich (2004), we propose four different areas of SRL: cognition, motivation, emotion and context. Cognition (including cognitive and metacognitive aspects) and motivation are the core areas of SRL that can be found in many SRL models (Panadero, 2017), such as in Pintrich’s model or in Boekaerts’s six component model of SRL. In addition, emotion is a relevant component of SRL models (Efklides, 2011; Panadero, 2017). Emotion has also become an increasingly important topic in recent years within SRL theory (Ben-Eliyahu, 2019) and in educational psychology research more generally (Pekrun, 2006). In health sciences education research, well-being, a concept related to emotion, is an important topic (Duffy et al., 2018; Fares et al., 2016; Frajerman et al., 2019). Therefore, in contrast to Pintrich, who combines the aspects of motivation and affect into one area, we integrate emotion as a separate component in our model. Finally, not only is context included in Pintrich’s framework, but the importance of context in terms of the learning environment has also been pointed out in health sciences education research (Berkhout et al., 2016; van Houten-Schat et al., 2018). Based on Pintrich’s SRL model, context is not seen as objective frameworks to which students are exposed. Rather, student take an active role. It is about how students interpret the context and about their ability to change the interpretation or, if possible, the context itself to reach their learning goals. We dropped the area behavior because, as also Pintrich (2000) pointed out, it overlaps with the area cognition.

We propose two different levels based on Nelson and Narens (1990) and, more specifically for SRL, on Boekaerts (1996) and Wirth et al. (2020): the learning-process level and the metalevel.^1^At the learning process level, students are in the middle of the learning process, consciously or unconsciously using cognitive strategies, experiencing different levels and aspects of motivation and emotions, and perceiving and interpreting the learning environment. At the metalevel, students step out of the learning process for a moment and reflect on their learning. The learning process level is similar to Boekaerts’s cognitive strategy-use-level and motivational beliefs as well as to Wirth and colleagues’ learning strategy layer. The metalevel is similar to Boekaerts’s goal level or Wirth and colleagues’ metacognitive layer. The metalevel is included in many SRL models but often refers only to the regulation of cognition, known as metacognition. Boekaerts suggests different levels of cognition and motivation, while Wirth and colleagues’ layers solely address the cognitive area.

**Fig. 1:**
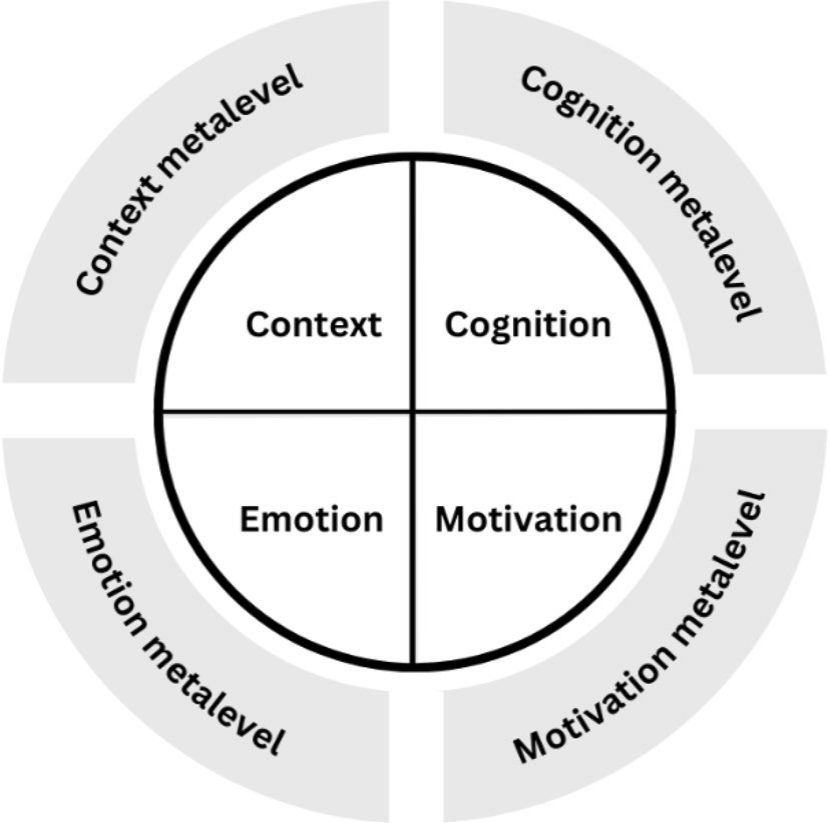
Eight workplace learning components are distinguished. The inner circle illustrates the learning process level components: The ideal learner implements appropriate learning strategies (cognition), is motivated (motivation), feels well (emotion) and perceives a supportive environment (context). The outer circle illustrates the metalevel components: The ideal learner steps out of the learning process and regulates cognition, motivation, emotion, and context

We extend our understanding of learning as both the learning process level and the metalevel address emotion and context in addition to cognition and motivation. We combine the four areas and two levels, which results in eight components (see Fig. 1). At the learning process level, (1) cognition refers to cognitive learning strategies such as rehearsal, organization or elaboration strategies (Weinstein et al., 2011); (2) motivation refers to motivational aspects as described by various motivation theories, such as expectancy-value theory (Eccles & Wigfield, 2020) and achievement goal theory (Urdan & Kaplan, 2020); (3) academic emotion refers to positive and negative emotions such as pride, enjoyment, frustration or anxiety as described, for example, in the control-value theory of achievement emotions (Pekrun, 2006) and in the Medical Emotion Scale (Duffy et al., 2018); and (4) context refers to the perception of the learning environment, including the physical and social environment (Strand et al., 2013).

At the metalevel, we refer to the components by using the terms ‘cognition metalevel’, ‘motivation metalevel’, ‘emotion metalevel’ and ‘context metalevel’. Following Pintrich (2004), we assume that the learner consciously or unconsciously anticipates, plans, monitors, adapts, evaluates and reacts not only in terms of cognition but also in terms of motivation, emotion and context. This assumption is also supported by research on motivation regulation and emotion regulation (Ben-Eliyahu, 2019; Wolters, 2003). There is evidence that cognitive, motivational and emotional processes on the metalevel are not distinct but share certain regulatory mechanisms (Kim et al., 2020; they did not consider metacontext).

In contrast to component-based models, process-based models emphasize the different phases of SRL. Zimmerman’s cyclical three-phase model (2008), which is often used in medical education (van Houten-Schat et al., 2018), differentiates among the forethought, performance and reflection phases. Therefore, the ideal learner analyses a task (e.g., planning) and addresses motivation and beliefs (e.g., self-efficacy) in the forethought phase. He or she monitors cognition, emotion and effort as well as task and environmental demands in the performance phase before assessing the achievement (e.g., strategic review) and reacting to it (e.g., rewards/sanctions) in the reflection phase.

### Measurement instruments

There is a wide range of methods for collecting data on the different components of learning, such as questionnaires, interviews, think-aloud techniques, learning diaries, or observations (Roth et al., 2016; Schunk & Greene, 2018; Wirth & Leutner, 2008). Self-report questionnaires are predominantly used to assess SRL in higher education (Roth et al., 2016). They allow for the assessment of core facets of learning that are not easily observable. They are also easier to administer than other methods, such as interviews or think-aloud protocols, especially in multivariate longitudinal studies. At the same time, the validity of the data produced by self-report questionnaires has been questioned (Wolters & Won, 2018), and there have been calls for the careful development of questionnaires (Gehlbach & Brinkworth, 2011).

For an overview of instruments and related scales in the field of SRL, primarily for the academic setting, see Roth et al. (2016). There are also self-report questionnaires specific to the metalevel components (Wolters, 1998), to emotion regulation in general (Burić et al., 2016; Loch et al., 2011) as well as to the workplace learning of health science students for emotion (Duffy et al., 2018) and for the learning environment (Roff & McAleer, 2017).

The established questionnaires mentioned above are not feasible in multivariate longitudinal studies of undergraduates’ workplace learning. They often address the academic setting and/or include long scales to cover a wide range of facets and to facilitate high validity. In multivariate longitudinal studies, long scales run the risk of overburdening participants (Hoerger, 2010). In addition, most instruments assess trait rather than state aspects of learning and may not be appropriate for examining change over time in longitudinal studies. Finally, when using questionnaires from different fields, such as motivation research or emotion research in multivariate studies, researchers often face the problem of construct contamination. This means that, for example, an emotion questionnaire includes as well items which address motivational aspects. In conclusion, there is a lack of self-report instruments that follow a more efficient approach with shorter scales, a focus on tracking changes over time, and distinct scales which would be appropriate for multivariate longitudinal psychological studies of workplace learning in undergraduate health sciences education.

In developing new scales with few items, different types of validity need to be thoroughly investigated. According to the American Educational Research Association et al. (2014) and Wolters and Won (2018), evidence of validity should be based on (1) content, (2) response processes, (3) internal structure, (4) relationships with other variables and (5) consequences of testing.

**Fig. 2:**
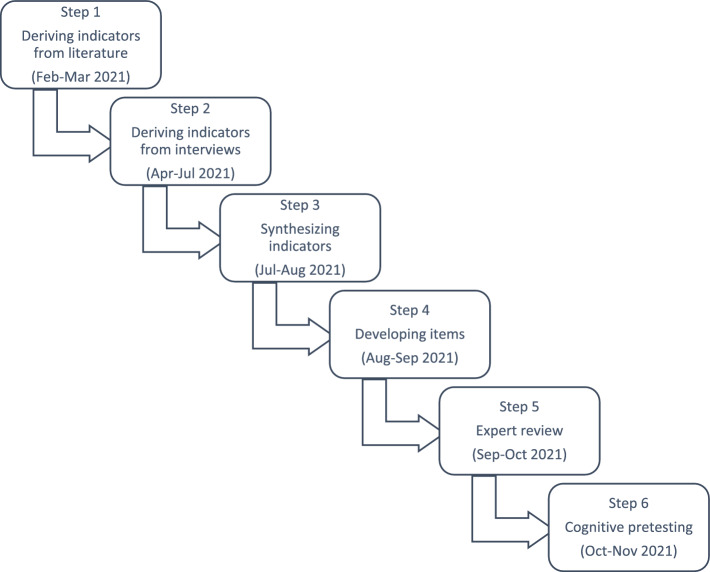
Method and the timeline of Study 1. The method is based on Gehlbach and Brinkworth (2011)

To address and ensure all types of validity, triangulation of methods is necessary when developing a self-report instrument. Gehlbach and Brinkworth (2011) recommend seven steps, from literature review to a pilot test of psychometric quality (see Fig. 2 for Step 1 to 6). Steps 1 to 6 can be summarized as the *qualitative part of questionnaire development*, addressing validity based on content and response processes. Step 7 is the *quantitative part of scale development*, addressing validity based on internal structure and relationships with other variables.

### Aim

The aim of the present study is to develop an instrument to assess different aspects of undergraduates’ workplace learning in health science education. Our aim is to provide a comprehensive inventory from which researchers can select those scales that are relevant to their research question, rather than using all the scales in one study. Each SRL-component should be represented by several indicators (scales), but each scale should contain only a few items to be applicable and reasonable in multivariate longitudinal studies.

We consider a medium degree of situational specificity (Roth et al., 2016) to be appropriate for our purpose. First, the inventory should be specific to a practical clinical setting (as opposed to an academic setting) of health sciences education but not to a specific field or profession. Second, the scales should assess a week of workplace learning but not specific days or situations. The inventory should be designed to capture changes over time; therefore, on the continuum between a state and trait measure, it should be more of a state measure (Geiser et al., 2017; Pekrun et al., 2018).

We follow Gehlbach and Brinkworth’s (2011) seven steps and conduct two studies. Study 1, a qualitative multimethod study, includes Steps 1 to 6 to develop the indicators and items. In Study 2, a quantitative study, we examine the psychometric properties of the scales.

## Study 1

The aim of Study 1 is to identify relevant indicators for each component of our model and to develop scales and items for undergraduate learning in the clinical practice setting of health sciences education. The inventory addresses four components at the learning process level, namely, cognition, motivation, emotion, and context, and four components at the learning metalevel, namely, the cognition metalevel, motivation metalevel, emotion metalevel, and context metalevel.

### Method

Figure 2 shows the steps and timeline of Study 1. To increase the trustworthiness of the process, each step was discussed by a multidisciplinary heterogeneous project team including SRL and health sciences education researchers, clinical teachers, and students. The project team met bi-weekly to ensure continuous discussion and decision making.

To identify relevant indicators and develop items, we considered the entire learning process of students throughout the day, from before they arrive in the workplace to their time in the workplace and after they leave. In the following sections, we describe our general process for developing indicators and items.

In Step 1, we identified relevant indicators for each component from the literature and from existing measures. We derived the indicators from the two most widely used SRL questionnaires in higher education (Roth et al., 2016). We added indicators from measures that were designed for the practical clinical setting (but not indicators that were too specific, such as those regarding surgeries) and that were specific to undergraduates (for emotion, we found only one relevant questionnaire; for context, the decision was based on the list provided by Roff and McAleer (2017). For metalevel motivation and emotion, we derived indicators from the most widely used questionnaires.

In Step 2, we identified indicators relevant to workplace learning for each component based on stakeholder statements. We conducted semistructured interviews with 6 students and 6 clinical educators (abbreviated as teachers) from German-speaking countries and with 6 researchers in the field of SRL and/or in health sciences education from different parts of Europe, Asia and North America. To ensure heterogeneity of perspectives, students and teachers were selected from six different health sciences institutions in three different countries based on recommendations from the respective offices of the vice-rectors for teaching and learning. All persons participated voluntarily, and only those who gave written consent participated. The interviews were conducted online and lasted approximately one to two hours per participant. The interview guideline started with explaining the topic, clarifying terms, and introductory questions. Participants were asked to describe helpful and detrimental aspects regarding emotion, motivation, learning strategies and perception of the context of workplace learning before, during and after undergraduates’ presence in workplace. Each theme was shortly introduced, followed by the question (E.g., ‘In the phase before learning at the workplace, which attitudes or beliefs are beneficial for motivation, and which are a hindrance?’). Finally, the meaning of the metalevel was explained (based on Pintrich, 2004), followed by the questions (E.g., ‘Is there anything here that is particularly important for successful learning? If so, what exactly?). The interviews were recorded and transcribed. The statements were categorized according to Mayring’s process flow of content structuring (2014). First, we defined the object of analysis. Second, we developed a theoretical-driven categorization system including definitions of categories. Third, we revised the categorization system as one worked through the material. Fourth, we coded the material. Finally, we reduced and summarized the extracted statements in each category. To enhance trustworthiness, the summaries were discussed and revised where necessary together with the whole project team over the course of a two-day project meeting.

In Step 3, we synthesized the list of indicators. Some indicators from Step 1 and Step 2 overlapped; in case of discrepancies in the categorization of indicators, we used the indicators from the interviews.

In Step 4, a scale with preliminary items was developed in German for each indicator. To take the students’ perspective into account, the formulation of the items was strongly based on the students’ statements derived from the interviews in Step 2.

In Step 5, the expert review, structured feedback on the preliminary items of the questionnaire was provided by experts who have relevant scientific publications in the field of SRL and/or in health sciences education. Nine researchers from German-speaking countries participated. The researchers were asked to review the indicators and the preliminary items for clarity, relevance and representation. Open-ended comments on each item were encouraged. The indicators and items were revised on the basis of the means of the relevance and clarity scores, the sums of the representativeness scores and the open-ended comments.

In Step 6, we carried out cognitive pretesting of the items. We interviewed potential respondents (students) to determine how they understood and responded to each item (Karabenick et al., 2007; Willis, 2015). We invited all students who currently were enrolled in courses in which they were learning in the clinical practical setting (approximately 350). In these courses, students rotate between different working environments and areas of veterinary medicine (e.g., anesthesia, surgery, reproduction medicine, imaging techniques, etc.). Approximately 20 students agreed to take part in the cognitive pretest and 14 students showed up. Seven students were in their 9th semester, and seven students were in their 11th semester. The students participated voluntarily, and only students who gave written consent participated. After an introduction, the students were asked to complete the questionnaire. We used reminded retrospective verbal probing (Willis, 2015) for each component: After each section (with items relating to one component), the students were asked to explain their cognitive process in answering the items. The interviewer took notes, and the interviews were recorded for documentation purposes. The interviews lasted approximately two hours, including one break. The indicators and items were revised based on the students’ comments.

Further information on the process of developing the indicators and items of each component can be found in the supplementary material.

### Results

The process of developing self-report measures for undergraduates’ workplace learning in health sciences education resulted in a comprehensive inventory. It includes several indicators for each of the four components at the learning process level in terms of cognition, motivation, emotion, and context and for each of the four components at the metalevel of learning in terms of cognition metalevel, motivation metalevel, emotion metalevel and context metalevel. The inventory comprises 31 indicators (= scales) and 159 items in total. Table 1 shows the indicators for the eight components, including definitions as well as the number of items per indicator and item examples.

**Table 1:** Results Study 1: Name of the component/sub-component/indicator (scale**)**, definition, itemexample, number of items and references

Component/sub-component/indicator (scale)	Definition	Itemexample	No. of items	References
**Cognition**	Cognition refers to learning strategies with a focus on workplace learning.			
*Cognitive learning strategies*	Cognitive learning strategies refer to the learning and practice of professional medical activities.			
Preparation	Preparation means activating knowledge as well as subject related preparation regarding professional medical activities before being in the clinical practice setting.	Before I came to the workplace, I worked to acquaint myself with relevant topics.	4	
Attention	Attention means focusing on and learning from performing or observing professional medical activities during being in the clinical practice setting.	At the workplace, I stayed concentrated while conducting practical medical tasks.	5	Schiefele and Wild (1994)
Rehearsal	Rehearsal means repeating and memorizing important facts and/or mentally playing through again important procedures during being in the clinical practice setting.	At the workplace, I consciously committed important information to memory.	5	Weinstein, Acee and Jung (2011)
Elaboration	Elaboration means integrating new information into one own’s information structur during being in the clinical practice setting.	At the workplace, I tried to connect the practical medical tasks to what I had previously learned.	5	Weinstein, Acee and Jung (2011)
Clarification	Clarification means clarifying unclear aspects or asking for support regarding professional medical activities that can be directly applied in the short run during learning in the clinical practice setting.	At the workplace, I asked for advice when something was unclear.	5	
Consolidation	Consolidation means processing experience and new knowledge regarding professional medical activities after learning in the clinical practice setting.	After leaving the workplace (no matter if e.g., 10 min or 2 h afterwards), I further deepened what I had learned and practiced.	5	
*Proximal metacognitive learning strategies*	Proximal metacognitive learning strategies are strategies, where students learn from regulating professional medical activities.			
Planning	Planning means anticipating and planning professional medical activities before being in the clinical practice setting.	Before I came to the workplace, I thought about what medical cases I could expect.	5	
Reviewing	Reviewing means to briefly pause during being in the clinical practice setting and think about if the professional medical activity (and related theoretical foundations and practical processes) are clear.	At the workplace, I recapitulated what I had practiced or learned in order to determine whether everything is clear to me.	5	
Reflection	Reflection means reflecting experience regarding clinical practical activities after learning in the clinical practice setting.	After leaving the workplace (no matter if e.g., 10 min or 2 h afterwards), I reflected on what I would do differently next time.	5	
**Motivation**	Motivation means instigating and sustaining goal-directed activity.			Schunk et al. (2014), Koenka (2020)
Expectancy of success	Expectancy of success means the individuals’ beliefs about how well they will do on an upcoming professional medical activity.	I am confident that this week I will be able to do what is asked of me.	5	Eccles and Wigfield (2020)
Situational interest	Interest means liking and willful engaging in practicing and learning.	This week I found the tasks interesting.	5	Schraw and Lehman (2001)
Mastery goal approach	Mastery approach goal orientation means focusing on attaining task-based or intrapersonal competence.	This week it was important to me to expand my knowledge.	5	Elliott, Murayama and Pekrun (2011)
Performance goal approach	Performance approach goal orientation means focusing on attaining normative competence.	This week it was important to me to practice exactly what the instructors are looking for when evaluating my performance.	5	Elliott, Murayama and Pekrun (2011)
Effort	Effort means persevering practicing and learning even when it is difficult.	This week I made an effort.	3	Items are based on Schiefele and Wild (1994)
Attention control (reverse coded)	Attention control means not getting distracted from practicing and learning.	This week I was not concentrated while practicing and studying.	3	Items are based on Schiefele and Wild (1994)
Proactive attitude	Proactive attitude means seeking and taking opportunities to practice and learn.	This week I took advantage of opportunities to gain hands-on practice.	5	
**Emotion**	Emotions are defined within the broader concept of affect but are distinguished from other affective phenomena, such as moods, in that emotions are more intense, have a clearer object-focus, a more salient cause, and are typically experienced for a shorter duration.			Scherer (2005), Shuman and Scherer (2014), Duffy et al. (2018)
Negative emotions	Negative emotions include fear/anxiety, frustration, shame, anger, confusion, disgust, disappointment, hopelessness, sadness and boredom.	Please think about how you felt this week. To what extent were you frustrated?	10	Items are based on Duffy et al. (2018)
Positive emotions	Positive emotions include pride, enjoyment, happiness, compassion, gratitude, curiosity, hope, relaxation and relief.	Please think about how you felt this week. To what extent were you proud?	9	Items are based on Duffy et al. (2018)
**Context**	Context means undergraduate medical students’ perceptions of multiple dimensions of the educational environment in the clinical practice setting.			Strand et al. (2013)
Organizational framework conditions	Organizational framework conditions mean students’ perceptions of the preparedness of the workplace and staff to integrate students.	I had the impression that the clinic / facility was well-organized, so that students encountered good contextual conditions.	5	Strand et al. (2013)
Supervisory quality	Supervisory quality means the students’ perceptions of learning environment shaped by the supervisor.	The instructors offered me opportunities to further develop.	6	
Staff support	Staff support means the students’ perceptions of learning environment shaped by the staff.	I was supported by members of the staff working here.	5	
Peer support	Staff support means the students’ perceptions of learning environment shaped by the peers.	I had the impression that the students support each other.	5	
Equal treatment	Equal treatment means the students’ perceptions of diversity culture.	All students were treated equally regardless of their gender.	4	Items are based on Strand et al. (2013)
**Cognition metalevel**	Metalevel for cognition means regulating the cognitive aspects of the learning process.			
Monitoring	Monitoring of cognitive aspects of the learning process means monitoring if the implemented (meta-) cognitive learning strategies are expedient.	This week I paid attention to whether my studying and practicing behavior would help me reach my goal.	5	
Control	Control of cognitive aspects of the learning process means changing (meta-) cognitive learning strategies in case of problems.	This week I changed the way I study or practice when I noticed that I was not getting better.	5	
**Motivation metalevel**	Metalevel for motivation means regulating the motivational aspects of the learning process.			
Monitoring	Monitoring of motivational aspects of the learning process means monitoring if the level and direction of motivation are expedient.	This week I paid attention to how motivated I am.	5	
Control	Control of motivational aspects of the learning process means changing the level and direction of motivation in case of problems.	This week I changed something when I noticed that I was not motivated.	5	
**Emotion metalevel**	Metalevel for emotion means regulating the emotional aspects of the learning process.			
Monitoring	Monitoring of emotional/affective aspects of the learning process means monitoring if the quality and intensity of emotions are expedient.	This week I reflected on my feelings while studying and practicing.	5	
Control	Control of emotional/affective aspects of the learning process means changing the quality and intensity of emotions in case of problems.	This week I changed something when I noticed that my feelings (e.g., fear or anger) were impeding me while studying or practicing.	5	
**Context metalevel**	Metalevel for context means regulating the contextual aspects of the learning process.			
Monitoring	Monitoring of contextual aspects of the learning process means monitoring if the learning environment is perceived as supportive.	This week I reflected on what contextual conditions^a^ accompany my studying and practicing. ^a^(organisational conditions, instructors, other students, on-site staff, equity concerns).	5	
Control	Control of contextual aspects of the learning process means adapting to difficult learning environment or changing contextual aspects in case of problems.	This week I changed how I study or practice in order to better adapt to contextual conditions. ^a^(organisational conditions, instructors, other students, on-site staff, equity concerns).	5	

All scales were administered using a five-point Likert scale with 1 = *does not apply at all*, 2 = *does not apply*, 3 = *partly applies*, 4 = *applies*, 5 = *fully applies;* for the ‘control’ scales at the metalevel, *6 = This case did not occur* was also included; except for the component emotion with 1 = *not at all*; 2 = *a little*; 3 = *moderately*; 4 = *fairly*; 5 = *very much*

## Study 2

The purpose of this study is to examine the psychometric properties of the scales developed in Study 1. Three aspects are examined in detail: (1) we examine whether the scales are unidimensional to provide evidence of validity based on internal structure; (2) we analyze the reliability of the scales; and (3) we examine whether the scales relate to other variables as theoretically expected by assessing the nomological network to provide evidence of validity based on relations with other variables, i.e., for convergent validity.

### Method

#### Participants

The results should be representative of a heterogeneous group of health science students in terms of cognition, motivation, emotion, and learning environment. We therefore decided to make an effort to reach the vast majority of a relevant cohort of students at one institution and thus obtain data from a heterogeneous group in terms of cognition, motivation, emotion, and learning environment, rather than send a questionnaire to different institutions and risk a biased sample by obtaining data from mostly motivated high achievers who feel good about their learning. Since the number of students from one institution only was insufficient for data analysis, we invited students from a second institution to participate. The target sample size was N = 200 based on a common rule of thumb for the minimum sample size when conducting confirmatory factor analysis (see Kline, 2016).

At Institution 1, the questionnaire was administered to the entire group of 200 students enrolled in a course in which students learn for the first time in the clinical practical setting over a relatively long period. This course is usually attended in the 9th semester. Students rotate between different work placements; thus, data were collected in heterogeneous workplace settings. Thirteen students did not give consent to their data being used for research purposes. Eleven participants had to be excluded from further analysis due to a high proportion of missing values (> 50%), resulting in a sample size of *n* = 176 at Institution 1.

At Institution 2, the questionnaire was sent via email to students in their practical year (usually in the 9^th^ and 10th semesters) in the winter semester of 2021/2022 (*n* **≈** 260). Students rotate between different work placements; thus, data were collected in heterogeneous workplace settings. The questionnaire was opened 91 times, but there were 38 responses, in which more than 50% of the items were completed. All 38 participants gave consent to their data being used for research purposes. Thus, combining both samples, the total sample size was *N* = 214 (78% female, 21% male, 1% diverse; age: 21 to 41 years; *M* = 24.79, *SD* = 2.74).

#### Measures

The newly developed inventory for workplace learning in health sciences education included 31 scales measuring eight components, namely, cognition, motivation, emotion, and context at the learning process level as well as the cognition metalevel, motivation metalevel, emotion metalevel and context metalevel (see Table 1). All scales were administered using a five-point Likert scale (1 = *does not apply at all*, 2 = *does not apply*, 3 = *partly applies*, 4 = *applies*, 5 = *fully applies*); for the ‘control’ scales at the metalevel, 6 = *This case did not occur* was also included). The ‘negative emotion’ and ‘positive emotion’ scales are special cases. The items were not newly developed but derived from the MES (Duffy et al., 2018). The response format established by Duffy et al. (2018) was used: 1 = *not at all*; 2 = *a little*; 3 = *moderately*; 4 = *fairly*; 5 = *very much*. Established measures were used to assess the nomological network. See Table 2 for details.

**Table 2:** Overview on established scales used in Study 2 including examples of items, number of items, and references

Component/scale	Item example	No. of Items	Reference
*Cognition*			
Organisation	I go through my notes and make an outline regarding the most important points.	3	Klingsieck, (2018)
Elaboration	I try to relate new concepts or theories to concepts or theories I already know.	3	
Critical Review	I wonder if the text I am working through is really convincing.	3	
Rehearsal	I memorize a self-made overview with the most important technical terms.	3	
Literature research	I look for further literature if certain topics are not yet completely clear to me.	3	
*Motivation*			
Self-efficacy	I am confident that I can understand even the most difficult material in course texts.	4	Kunter et al. (2002)
Learning goal approach	I participate in courses because I want to learn new things.	4	Schwarzer and Jerusalem (1999)
Performance goal approach	In my studies, I strive to be better than the others.	4	
Performance goal avoidance	In my studies, I make sure that others don't think I'm stupid.	4	
Attention control	It is difficult for me to stay on task.	3	Boerner et al. (2005)
*Emotion*			
Negative Emotions	To what extent were you feeling frustrated.	10	Duffy et al. (2018)
Positive Emotions	To what extent were you feeling proud.	9	
*Context*			
Perception of teachers	The teachers are good at providing feedback to students.	11	Rotthoff et al. (2010, 2011)
Perception of atmosphere	The atmosphere during lectures is relaxed.	12	
*Cognition metalevel*			
Goalsetting/planning	I am clear about what my goals are when learning.	6	Boerner et al. (2005)
Control	To identify gaps in my knowledge, I recap the most important content without using my notes to assist me.	6	
Regulation	If I realize that I'd better learn something else first, I change the sequence accordingly.	8	
*Motivation metalevel*			
Increasing situational interest	I make learning more enjoyable by trying to approach it in a playful way.	5	Schwinger et al. (2007)
Increasing personal value	I look for connections between the task material and the rest of my life.	3	
Performance-goal-approach oriented self-instruction	I make myself aware of the importance of getting good grades/evaluations.	5	
Self-rewarding	I tell myself that if I keep working for now, I can do something nice after I finish the job.	4	
Mastery-goal-approach oriented self-instruction	I persuade myself to work harder for the sake of learning.	4	
Controlling learning environment	I deliberately choose times to study when I can concentrate particularly well.	3	
Performance-goal-avoidance oriented self-instruction	I tell myself that I have to try harder if I don't want to embarrass myself.	3	
Setting subgoals	I divide the work into small sections so that I feel I can manage it more easily.	3	
*Emotion metalevel*			
Self-incrimination (Self-blame)	I feel that I am the one to blame for it.	3	Loch et al. (2011) Garnefski et al. (2001)
Acceptance	I think that I have to accept that this has happened.	3	
Rumination	I often think about how I feel about what I have experienced.	3	
Positive refocusing	I think of nicer things than what I have experienced.	3	
Refocus(ing) on planning	I think of what I can do best.	3	
Positive reevaluation (reappraisal)	I think that I can become a stronger person as a result of what has happened.	3	
Relativize (putting into perspective)	I think that other people go through much worse experiences.	3	
Catastrophize	I keep thinking about how terrible it is what I have experienced.	3	
Accusing (blaming) others	I feel that others are to blame for it.	3	
*Context metalevel*			
No appropriate scales available			

Item examples and response formats were slightly adapted from original questionnaires where necessary (e.g. ‘At school’ was replaced by ‘In my studies’). To not overextend students, all scales were administered using the same five-point Likert scale with 1 = *does not apply at all*, 2 = *does not apply*, 3 = *partly applies*, 4 = *applies*, 5 = *fully applies;* except for the component emotion with 1 = *not at all*; 2 = *a little*; 3 = *moderately*; 4 = *fairly*; 5 = *very much*

#### Procedure

At Institution 1, the questionnaires were completed as part of the course and supported the course learning goal of “reflecting on one’s own learning and practice”. Data collection was spread over a week (either from 6 to 10th December 2021 or from 13 to 17th December 2021) using the online survey tool unipark© (EFS Survey, 2022). Most of the established scales were more trait-like measures and were presented at the beginning of the week while most of the newly developed scales were presented at the end of the week.

At Institution 2, the rectorate invited all students currently in their practical year. Students received a link to the survey that comprised all questionnaires. They were allowed to pause and continue filling in the questionnaire later between 6 and 17th December 2021 using the online survey tool unipark© (EFS Survey, 2022).

#### Data analysis

To assess unidimensionality, confirmatory factor analysis (CFA) was used: a one-factor model based on all items of the scale was specified for each scale, using the software Mplus 8.6 (Muthen & Muthen, 1998–2017). Full information maximum likelihood method was used to deal with missing data (Enders, 2022). Model fit was assessed using fit indices based on conventional cut-off criteria for an acceptable model fit, i.e., CFI and TLI ≥ 0.90 and RMSEA and SRMR ≤ 0.08. In the case of poor model fit, residual covariances resulting from similarities in item meaning were specified (Bandalos, 2021). In addition, standardized factor loadings were used to identify and exclude items of low psychometric quality to further improve model fit. The ‘negative emotion’ and ‘positive emotion’ scales are special cases. By performing a CFA, we aimed to identify the most relevant emotions and to provide a short version of these MES scales within this questionnaire. We also did not analyze the ‘effort’ and ‘attention control’ scales because the items were reformulated with very small changes from the established scales on academic learning (Klingsieck, 2018).

To assess reliability, McDonald's composite reliability coefficient ω (1970) was calculated for each scale. Acceptable reliability is indicated by ω ≥ 0.70. To assess the nomological network and thus to investigate whether our newly developed scales were related to the established scales as theoretically expected, we used correlations.

### Results

#### Unidimensionality and reliability

After a total of four items were excluded, the CFA model fit was acceptable and indicated the unidimensionality of all scales. Exceptions included the ‘positive emotion’ and ‘negative emotion’ scales, which are special cases. The aim was to provide a short version of these established scales. Based on the results of the interviews in Step 2 of Study 1 in combination with the factor loadings, we excluded five out of nine items of the ‘positive emotion’ scale and six of eleven items of the ‘negative emotion’ scale. The CFA of the short scales showed acceptable model fit, indicating the unidimensionality of the two scales. The omega values of all scales were within the acceptable range, indicating acceptable reliability. See Table 3 on CFA/reliability details. The final questionnaire with all scales and items can be found at the end of the document in Table 5.

**Table 3:** Unidimensionality and reliability

Component/sub-component/scale	No. of items	CFA						Range of Std. factor loadings	McDonald’s omega
		Chi^2^	df	CFI	TLI	RMSEA	SRMR		
**Cognition**									
*Cognitive learning strategies*									
Preparation	4	0.059	2	1	1	0	0.002	0.59–0.80	0.867
Attention	5	0.059	2	1	1	0	0.002	0.25–0.86	0.891
Rehearsal	5	9.665	5	0.982	0.963	0.066	0.035	0.34–0.83	0.787
Elaboration	5	0.905	4	1	1	0	0.009	0.46–0.91	0.753
Clarification	5	8.316	4	0.983	0.957	0.071	0.021	0.33–0.86	0.713
Consolidation	5	10.578	5	0.985	0.969	0.072	0.025	0.57–0.82	0.859
*Proximal metacognitive learning strategies*									
Planning	5	5.498	3	0.988	0.960	0.062	0.028	0.50–0.84	0.695
Reviewing*	4	2.716	2	0.993	0.978	0.041	0.021	0.47–0.77	0.693
Reflection*	4	3.341	2	0.993	0.979	0.056	0.022	0.63–0.87	0.818
**Motivation**									
Expectancy of success	5	7.746	4	0.993	0.982	0.066	0.016	0.76–0.86	0.895
Situational interest	5	2.452	4	1	1	0	0.007	0.73–0.90	0.883
Mastery goal approach	5	5.881	5	0.998	0.997	0.029	0.013	0.80–0.88	0.923
Performance goal approach	5	6.931	4	0.992	0.980	0.059	0.025	0.51–0.88	0.828
Effort	3	/	/	/	/	/	/	0.48–0.87	0.659
Attention control	3	/	/	/	/	/	/	0.73–0.86	0.842
Proactive attitude*	4	0.314	1	1	1	0	0.003	0.40–0.91	0.804
**Emotion**									
Negative emotion	4	1.97	2	1.000	1.000	0.000	0.018	0.62–0.82	0.792
Positive emotions	4	1.06	2	1.000	1.000	0.000	0.010	0.67–0.73	0.788
**Context**									
Organizational framework	5	4.011	5	1	1	0	0.017	0.61–0.90	0.874
Supervisory quality	6	19.439	9	0.976	0.96	0.075	0.03	0.42–0.83	0.849
Staff support	5	8.075	4	0.985	0.963	0.070	0.021	0.71–0.85	0.859
Peer support	5	2.278	3	1	1	0	0.009	0.58–0.82	0.855
Equal treatment*	3	/	/	/	/	/	/	0.40–0.89	0.761
**Metalevel cognition**									
Monitoring	5	6.049	5	0.997	0.995	0.032	0.017	0.71–0.88	0.914
Control	5	8.595	5	0.971	0.941	0.065	0.035	0.75–0.80	0.837
**Metalevel motivation**									
Monitoring	5	2.646	4	1	1	0	0.016	0.71–0.85	0.881
Control	5	5.521	3	0.980	0.933	0.069	0.021	0.47–0.81	0.752
**Metalevel emotion**									
Monitoring	5	2.304	5	1	1	0	0.006	0.77–0.93	0.929
Control	5	1.426	3	1	1	0	0.020	0.32–0.83	0.687
**Metalevel context**									
Monitoring	5	8.314	5	0.993	0.985	0.056	0.017	0.68–0.91	0.924
Control	5	7.791	5	0.985	0.971	0.058	0.031	0.40–0.81	0.868

Scales including three items only were not part of the analysis due to low number of items. They are adapted versions from established scales. The scales marked with * show the final results of analysis after excluding one item per scale due to low factor loadings

#### Nomological network

The nomological network was analyzed by assessing the relationship between the newly developed scales and the corresponding established scales. Please see Table 4 for the respective correlation coefficients.

**Table 4:** Nomological network: correlations between related constructs

Cognitive learning strategies	Organisation	Elaboration	Critical review	Rehearsal	Literature research				
Preparation	**0.163**	**0.167**	**0.239**	0.087	**0.320**				
Attention	0.111	**0.288**	0.105	− 0.059	**0.208**				
Rehearsal	0.145	**0.266**	**0.208**	**0.054**	**0.263**				
Elaboration	**0.179**	**0.357**	**0.264**	**− 0.121**	**0.209**				
Clarification	0.073	**0.318**	**0.244**	**− 0.167**	**0.361**				
Consolidation	**0.212**	**0.210**	**0.252**	0.068	**0.231**				

Proximal metacognitive learning strategies	Goalsetting/planning	Control	Regulation						
Planning	0.145	0.075	0.065						
Reviewing	0.096	0.165	**0.183**						
Reflection	0.079	0.151	0.047						

Motivation	Self-efficacy	Mastery goal approach	Performance goal appoach	Performance goal avoidance	Attention control				
Expectancy of success	**0.463**	**0.376**	0.051	− 0.016	− 0.126				
Situational interest	0.054	**0.269**	0.070	0.094	− 0.071				
Mastery goal approach	0.130	**0.401**	0.067	0.066	− 0.142				
Performance goal approach	− 0.115	0.096	**0.242**	**0.271**	0.035				
Effort	0.036	0**.379**	− 0.041	0.011	− 0.086				
Attention control	− 0.207	− 0.113	− 0.003	0.061	**0.312**				
Proactive attitude	0.131	0.360	0.058	− 0.008	− 0.059				

Context	Perception of teachers	Perception of atmosphere							
Organizational framework conditions	**0.707**	**0.742**							
Supervisory quality	**0.684**	**0.701**							
Staff support	**0.715**	**0.720**							
Peer support	**0.537**	**0.511**							
Equal treatment	0.465	0.282							

Cognition Metalevel	Goalsetting/ Planning	Control	Regulation						
Monitoring	**0.183**	0.146	**0.284**						
Control	0.121	0.138	0.208						

Motivation metalevel	Increasing situational interest	Increasing personal value	Performance-goal-approach oriented self-instruction	Self-rewarding	Mastery-goal-approach oriented self-instruction	Controlling learning environment	Performance-goal-avoidance oriented self-instruction	Setting subgoals	
Monitoring	**0.199**	0.148	0.162	0.134	**0.285**	**0.271**	**0.194**	0.075	
Control	0.187	0.149	0.082	0.164	0.321	0.264	− 0.021	0.149	

Emotion metalevel	Self-incrimination	Acceptance	Rumination	Positive refocusing	Refocusing on planning	Positive reevaluation	Relativize	Catastrophize	Accusing others
Monitoring	0.139	0.161	**0.414**	0.121	**0.266**	0.157	0.028	**0.182**	0.013
Control	− 0.070	0.190	0.169	0.074	0.197	0.273	0.183	− 0.063	− 0.069

Significant correlation results are shown in bold

## Discussion

In the current study, we developed an inventory for assessing undergraduates’ workplace learning in health sciences education. To ensure validity, a thorough multimethod approach was undertaken involving students, teachers, SRL researchers and health sciences researchers in the field (Gehlbach & Brinkworth, 2011). We conducted two studies, with Study 1 representing the qualitative part of the development process and Study 2 representing the quantitative analysis of the psychometric properties of the scales. The studies yielded a comprehensive set of 31 scales addressing four different areas, namely, cognition, motivation, emotion, and context, at two different levels, namely, the learning process level and the metalevel, resulting in eight components. Each component is represented by several short scales so that the administration of the scales is feasible in the practice setting. In the following, the results are discussed separately for each component, starting with learning process level components and continuing with metalevel components.

### Learning process level

At the learning process level, we included the cognition, motivation, emotion, and context components. At this level, students use cognitive learning strategies, experience different aspects and levels of motivation and emotion, and perceive and interpret the workplace context.

#### Cognition

The cognition component refers to learning strategies with a focus on workplace learning, i.e., learning and practicing professional medical activities. The ideal student anticipates the day as far as possible and acquires knowledge by *preparing* himself or herself and by *planning* the medical activities ahead. In the workplace, he or she acquires knowledge and skills by *paying attention*, *rehearsing* and *elaborating.* While in the workplace, the ideal student *reviews* whether he or she understands the medical procedures and *clarifies* unclear points. After being in the workplace, the ideal student *consolidates* his or her knowledge and *reflects* on his or her professional medical performance. The mentioned strategies are divided into cognitive learning strategies, and proximal metacognitive learning strategies, (see Table 5) and represent the whole learning process of a learning day: before, during and after students’ presence in the clinical practice setting. Psychometric analysis indicated the unidimensionality and acceptable reliability of all scales.

**Table 5:** The Workplace Learning Inventory in Health Sciences Education

Component/sub-component/scale	Item label	German language		English translation	
**Cognition**					
*Cognitive learning strategies*					
Preparation	Pre1	Bevor ich in die Klinik bzw. in den Betrieb kam, habe ich …	… mich in relevante Themen eingearbeitet	Before I came to the workplace, …	… I worked to acquaint myself with relevant topics
	Pre2		… mich inhaltlich auf medizinische Fälle oder Themen vorbereitet		… I prepared substantively for medical cases and topics
	Pre3		… mich mit ausgewählten Themen beschäftigt		… I engaged with selected topics
	Pre4		… mir in Erinnerung gerufen, was ich zu den anstehenden medizinischen Fällen oder Themen schon weiß		… I actively recalled what I already know about the upcoming medical cases or topics
Attention	Att1	In der Klinik/ Im Betrieb …	… war ich bei der Durchführung von medizinisch-praktischen Tätigkeiten konzentriert	At the workplace, …	… I stayed concentrated while conducting practical medical tasks
	Att2		… war ich bei medizinisch-praktischen Tätigkeiten voll und ganz bei der Sache		… I kept completely on task during practical medical tasks
	Att3		… war ich bei medizinisch-praktischen Tätigkeiten gedanklich präsent		… I was mentally present during practical medical tasks
	Att4		… habe ich andere bei der Durchführung von medizinisch-praktischen Tätigkeiten aufmerksam beobachtet		… I attentively observed others while they completed practical medical tasks
	Att5		… war ich bei medizinisch-praktische Tätigkeiten fokussiert		… I stayed focused during practical medical tasks
Rehearsal	Reh1	In der Klinik/ Im Betrieb …	… habe ich mir Wichtiges bewusst eingeprägt	At the workplace, …	… I consciously committed important information to memory
	Reh2		… habe ich komplexe Abläufe in Gedanken nochmals durchgespielt, um sie mir zu merken		… I went through complex procedures again in my mind in order to make note of them
	Reh3		… habe ich wichtige Aspekte auswendig gelernt		… I memorized important aspects
	Reh4		… habe ich mir Abläufe bewusst eingeprägt		… I consciously committed procedures to memory
	Reh5		… habe ich mir Neues bewusst gemerkt		… I consciously took note of new information
Elaboration	Ela1	In der Klinik/ Im Betrieb …	… habe ich versucht, die medizinisch-praktischen Tätigkeiten mit dem, was ich bisher gelernt habe, zu verbinden	At the workplace, …	… I tried to connect the practical medical tasks to what I had previously learned
	Ela2		… habe ich mit anderen über meine medizinisch-praktischen Erfahrungen diskutiert		… I discussed my practical medical experiences with others
	Ela3		… habe ich meine neuen Erfahrungen mit bisherigen Erfahrungen in Verbindung gebracht		… I connected my new experiences with my previous ones
	Ela4		… habe ich meine praktischen Erfahrungen mit theoretischem Wissen verknüpft		… I linked my practical experiences with theoretical knowledge
	Ela5		… habe ich überlegt, in welchen Fällen ich das Geübte oder Gelernte benötigen werde		… I thought about in what cases I would need to apply what I had practiced or learned
Clarification	Cla1	In der Klinik/ Im Betrieb …	… habe ich bei Unklarheiten um Rat gefragt	At the workplace, …	… I asked for advice when something was unclear
	Cla2		… habe ich offene Fragen noch vor Ort geklärt		… I clarified my remaining questions then and there
	Cla3		… habe ich mir Unklares noch einmal erläutern lassen		… I had things that were unclear explained to me again
	Cla4		… habe ich andere bei Bedarf um Tipps und Tricks gebeten		… I asked others for tips and tricks as needed
	Cla5		…habe ich bei Bedarf zu bestimmten Themen nachgelesen		… I read up on certain topics as needed
Consolidation	Con1	Nach Verlassen der Klinik bzw. des Betriebes (egal ob z.B. 10 min oder 2 h danach), habe ich …	… das, was ich gelernt und geübt habe, nochmals vertieft	After leaving the workplace (not matter if e.g., 10 min or 2 h afterwards), …	… I further deepened what I had learned and practiced
	Con2		… noch etwas nachgelesen		… I did some further reading up
	Con3		… mir Notizen gemacht		… I took notes
	Con4		… Wichtiges nochmal eingeprägt		… I committed important information once again to memory
	Con5		… Relevantes nochmal wiederholt		… I reviewed relevant information once again
*Proximal metacognitive learning strategies*					
Planning	Pla1	Bevor ich in die Klinik bzw. in den Betrieb kam, habe ich …	… überlegt, welche medizinischen Fälle mich erwarten	Before I came to the workplace, …	… I thought about what medical cases I could expect
	Pla2		… überlegt, welche fachlichen Themen heute relevant sein werden		… I thought about what substantive topics will be relevant today
	Pla3		… überlegt, welche Lern- und Übungsmöglichkeiten sich ergeben könnten		… I thought about what opportunities for learning and practice might arise
	Pla4		… überlegt, was ich an diesem Tag lernen oder üben möchte		… I thought about what I would like to learn or practice today
	Pla5		… überlegt, wie der Tag ablaufen könnte		… I thought about how the day might go
Reviewing	Rev1	Vor Ort in der Klinik bzw. im Betrieb…	… habe ich das Geübte oder Gelernte rekapituliert, um festzustellen, ob mir alles klar ist	At the workplace, …	… I recapitulated what I had practiced or learned in order to determine whether everything is clear to me
	Rev2		… habe ich überlegt, ob ich alles verstehe		… I reflected on whether I understand everything
	Rev3		… habe ich innegehalten, um zu überlegen was ich noch üben oder lernen soll		… I went inside myself to reflect on what I should still practice or learn
	Rev4		… habe ich es ignoriert, wenn mir etwas nicht ganz klar war		… I ignored whenever something was not completely clear to me
Reflection	Ref1	Nach Verlassen der Klinik bzw. des Betriebes (egal ob z.B. 10 min oder 2 h danach), habe ich …	… nachgedacht, was ich nächstes Mal anders machen würde	After leaving the workplace (no matter if e.g., 10 min or 2 h afterwards), …	… I reflected on what I would do differently next time
	Ref2		… nachgedacht, was gut geklappt hat		… I reflected on what had worked well
	Ref3		… nachgedacht, was meine Stärken und Schwächen sind		… I reflected on what my strengths and weaknesses are
	Ref4		… nachgedacht, was ich noch lernen oder üben muss		… I reflected on what I still need to learn or practice
**Motivation**					
Expectancy of success	EoS1	Ich bin zuversichtlich, dass ich diese Woche …	… das, was gefordert wird, umsetzen kann	I am confident that this week I…	… will be able to do what is asked of me
	EoS2		… auch herausfordernde Situationen meistern werde		… will be able to successfully handle even challenging situations
	EoS3		… auch anspruchsvolle Tätigkeiten schaffen werde		… will be able to successfully complete even demanding tasks
	EoS4		… den Anforderungen gerecht werden kann		… will be able to meet requirements
	EoS5		… die Aufgaben erfüllen kann		… will be able to complete the assigned tasks
Situational interest	SiI1	Diese Woche …	… habe ich die Aufgaben interessant gefunden	This week…	… I found the tasks interesting
	SiI2		… hat mich der Fachbereich interessiert		… I was interested in the clinical area
	SiI3		… waren die Inhalte für mich von Interesse		… the content was interesting to me
	SiI4		… fand ich die medizinischen Fälle interessant		… I found the medical cases interesting
	SiI5		… war die Arbeit vor Ort spannend		… the on-site work was exciting
Mastery approach	MaA1	Diese Woche…	… war es mir wichtig, mein Wissen zu erweitern	This week…	… it was important to me to expand my knowledge
	MaA2		… war es mir wichtig, meine Kompetenzen stetig zu verbessern		… it was important to me to constantly improve my competences
	MaA3		… war es mir wichtig, etwas Neues zu erfahren		… it was important to me to experience something new
	MaA4		… war es mir wichtig, medizinisch-praktische Erfahrung zu sammeln		… it was important to me to gain practical medical experience
	MaA5		… war es mir wichtig, Verständnis in diesem Fachbereich zu entwickeln		… it was important to me to develop an understanding of this clinical area
Performance approach	PeA1	Diese Woche…	… war es mir wichtig, dass ich genau das lerne, was Lehrende von mir erwarten	This week…	… it was important to me to learn exactly what the instructors expect of me
	PeA2		… war es mir wichtig, dass ich mich auf das konzentriere, was für eine gute Beurteilung von Lehrenden gefordert ist		… it was important to me to concentrate on what the instructors require for a good evaluation
	PeA3		… war es mir wichtig, genau das zu üben, worauf es Lehrenden bei der Beurteilung meiner Leistung ankommt		… it was important to me to practice exactly what the instructors are looking for when evaluating my performance
	PeA4		… war es mir wichtig, vor Lehrenden eine gute Leistung zu zeigen		… it was important to me to demonstrate good performance to the instructors
	PeA5		… war es mir wichtig, vor Lehrenden gut dazustehen		… it was important to me to be seen by the instructors in a good light
Effort	Eff1	Diese Woche…	… habe ich mich angestrengt	This week…	… I made an effort
	Eff2		… habe ich nicht aufgegeben, auch wenn es schwierig wurde		… I did not give up even when things got difficult
	Eff3		… habe ich außerhalb der Klinik bzw. des Betriebes gelernt, wenn es sein musste		… I studied outside the workplace when necessary
Attention control	AtC1	Diese Woche…	… war ich beim Üben und Lernen unkonzentriert	This week…	… I was not concentrated while practicing and studying
	AtC2		… fiel es mir schwer, beim Üben und Lernen bei der Sache zu bleiben		… I found it difficult to keep on task while practicing and studying
	AtC3		… war ich beim Üben und Lernen leicht abzulenken		… I was easily distracted while practicing and studying
Proactive attitude	PrA1	Diese Woche…	… habe ich Möglichkeiten zum praktischen Üben genutzt	This week…	… I took advantage of opportunities to gain hands-on practice
	PrA2		… habe ich Gelegenheiten, etwas selber auszuprobieren, ergriffen		… I took advantage of chances to try something out myself
	PrA3*		…habe ich es nach Möglichkeit vermieden, praktische Tätigkeiten selber durchzuführen		… I tried to avoid carrying out practical tasks myself when possible
	PrA4		… habe ich die sich mir bietenden Chancen, praktische Tätigkeiten auszuprobieren, genutzt		… I took advantage of the chances offered to me to try out practical tasks
**Emotion**					
Negative emotions	NeE1	Bitte denken Sie daran, wie Sie sich diese Woche gefühlt haben. Inwieweit waren Sie …	… ängstlich	Please think about how you felt this week. To what extent were you.	… anxious
	NeE2		… frustriert		… frustrated
	NeE3		… verärgert		… angry
	NeE4		… traurig		… sad
Positive emotions	PoE1	Bitte denken Sie daran, wie Sie sich diese Woche gefühlt haben. Inwieweit waren Sie …	… stolz	Please think about how you felt this week. To what extent were you …	… proud
	PoE2		… glücklich		… happy
	PoE3		… hoffnungsvoll		… hopeful
	PoE4		… neugierig		… curious
**Context**					
Organizational framework conditions	Ofc1	Ich hatte den Eindruck, …	… dass die Klinik bzw. der Betrieb gut organisiert war, so dass Studierende gute Rahmenbedingungen vorfanden	This week I had the impression …	… that the clinic / facility was well-organized, so that students encountered good contextual conditions
	Ofc2		… dass die Klinik bzw. der Betrieb ausreichende Ressouren, im Sinne von Literatur oder Zugang zu Datenbanken, für die Studierenden zur Verfügung stellt		… that the clinic / facility made sufficient resources in terms of literature or database access available to students
	Ofc3		… dass das Team, das hier arbeitet, auf die Studierenden vorbereitet war		… that the staff working here were prepared for the students
	Ofc4		… dass die Lehrenden ausreichend Zeit für die Betreuung der Studierenden hatten		… that the instructors had sufficient time to supervise the students
	Ofc5		… dass die Klinik bzw. der Betrieb räumlich auf die Studierenden vorbereitet war (z.B. Rückzugsräume, Arbeitsplätze)		… that the clinic / facility was prepared for the students in spatial terms (e.g. rooms for rest and relaxation, workspaces)
Supervisory quality	SuQ1	Bitte denken Sie an diese Woche:	Die Lehrenden boten mir Gelegenheiten, mich weiterzuentwickeln	This week…	… the instructors offered me opportunities to further develop
	SuQ2		Die Lehrenden wollten mir wirklich etwas beibringen		… the instructors really wanted to teach me something
	SuQ3		Die Lehrenden kennen sich auf ihrem Gebiet gut aus		… the instructors are well-versed in their discipline
	SuQ4		Bei diesen Lehrenden konnte ich neue Erfahrungen sammeln		… I was able to have new experiences with these instructors
	SuQ5		Die Lehrenden haben mich dazu angeregt, über die Art und Weise, wie ich lerne, nachzudenken		… the instructors encouraged me to reflect on the way I learn
	SuQ6		Die Lehrenden haben mich positiv motiviert		… the instructors motivated me in a positive way
Staff support	StS1	Bitte denken Sie an diese Woche:	Ich hatte Unterstützung von Personen aus dem Team, das hier arbeitet	This week …	… I was supported by members of the staff working here
	StS2		Ich konnte mich bei Problemen an Personen aus dem Team, das hier arbeitet, wenden		… I could turn to members of the staff working here when problems arose
	StS3		Ich habe mich von den Personen aus dem Team, das hier arbeitet, willkommen gefühlt		… I felt welcomed by the members of the staff working here
	StS4		Ich konnte mich mit Personen aus dem Team, das hier arbeitet, austauschen		… I could exchange experiences and views with members of the staff working here
	StS5		Ich habe mich von Personen aus dem Team, das hier arbeitet, wertgeschätzt gefühlt		… I felt appreciated by members of the staff working here
Peer support	PeS1	Bitte denken Sie an diese Woche:	Ich hatte den Eindruck, dass sich die Studierenden gegenseitig unterstützen	This week …	… I had the impression that the students support each other
	PeS2		Ich hatte den Eindruck, dass die Studierenden Rücksicht darauf nehmen, dass jede/r Gelegenheit zum praktischen Üben bekommt		… I had the impression that the students take care to ensure that everyone has the opportunity for hands-on practice
	PeS3		Ich fühlte mich in die Gruppe der Studierenden eingebunden		… I felt integrated into the group of students
	PeS4		Ich konnte mich mit Mitstudierenden über meine Erfahrungen austauschen		… I could discuss my experiences with other students
	PeS5		Bei Problemen konnte ich mich an Mitstudierende wenden		… I could turn to other students when problems arose
Equal treatment	EqT1	Bitte denken Sie an diese Woche:	Alle Studierenden wurden unabhängig von ihrem Geschlecht gleich behandelt	This week …	… all students were treated equally regardless of gender
	EqT2		Alle Studierenden wurden unabhängig von ihrem kulturellen Hintergrund gleich behandelt		… all students were treated equally regardless of cultural background
	EqT3		Ich konnte sexistische Diskriminierung beobachten.*		… I observed sex discrimination
**Cognition metalevel**					
Monitoring	CoM1	Diese Woche…	… habe ich darauf geachtet, ob mein Lern- und Übungsverhalten zielführend ist	This week…	… I paid attention to whether my studying and practicing behavior would help me reach my goal
	CoM2		… habe ich darauf geachtet, ob ich mit meinem Lernen und Üben zufrieden bin		… I paid attention to whether I am satisfied with my studying and practicing
	CoM3		… habe ich darauf geachtet, ob ich Lern- oder Übungsmöglichkeiten sinnvoll nutze		… I paid attention to whether I am taking good advantage of opportunities for studying and practicing
	CoM4		… habe ich darauf geachtet, ob ich effektiv bin in der Art und Weise, wie ich lerne und übe		… I paid attention to whether my way of studying and practicing is effective
	CoM5		… habe ich darauf geachtet, ob die Art und Weise, wie ich lerne oder übe, sinnvoll ist		… I paid attention to whether the way I study or practice makes sense
Control	CoC1	Diese Woche…	… habe ich die Art und Weise, wie ich lerne oder übe, geändert, wenn ich bemerkt habe, dass ich nicht besser werde		… I changed the way I study or practice when I noticed that I was not getting better
	CoC2		… habe ich überlegt, was ich ausprobieren könnte, wenn mein Lernen und Üben nicht erfolgreich war	This week…	… I thought about what I could try out if my learning and practicing was not successful
	CoC3		… habe ich mich mit anderen über die Art und Weise, wie man lernen oder üben kann, unterhalten, wenn ich bemerkt habe, dass ich Probleme habe		… I talked to others about how to study or practice when I noticed that I was having problems
	CoC4		… habe ich darüber nachgedacht, was ich an der Art und Weise, wie ich lerne und übe, noch verbessern kann, wenn ich unzufrieden war		… I reflected on what I could still improve about how I study and practice when I was dissatisfied
	CoC5		… habe ich mein Vorgehen beim Lernen oder Üben geändert, wenn ich bemerkt habe, dass mein bisheriges Vorgehen nicht zum Ziel führt		… I changed how I approach studying or practicing when I noticed that my previous approach was not helping me reach my goal
**Motivation metalevel**					
Monitoring	MoM1	Diese Woche…	… habe ich darauf geachtet, wie motiviert ich bin	This week…	… I paid attention to how motivated I am
	MoM2		... habe ich darauf geachtet, was mich zum Lernen und Üben motiviert		… I paid attention to what motivates me to study and practice
	MoM3		… habe ich darauf geachtet, ob ich motiviert bin		… I paid attention to whether I am motivated.
	MoM4		… habe ich darauf geachtet, was mich demotiviert		… I paid attention to what demotivates me
	MoM5		… habe ich darauf geachtet, dass ich mir meiner Motivation bewusst bin		… I ensured that I was aware of my level of motivation
Control	MoC1	Diese Woche…	… habe ich etwas geändert, wenn ich gemerkt habe, dass ich nicht motiviert bin	This week…	… I changed something when I noticed that I was not motivated
	MoC2		… habe ich überlegt, wie ich mit mangelnder Motivation umgehe		… I thought about how to deal with a lack of motivation
	MoC3		… konnte ich mich motivieren, wenn ich bemerkt habe, dass mir die Motivation fehlt		… I was able to motivate myself when I noticed that I lacked motivation
	MoC4		… konnte ich mich zum Lernen und Üben aufraffen, auch wenn ich demotiviert war		… I was able to bring myself to study or practice, even when I lacked motivation
	MoC5		… habe ich überlegt, auf welche Art und Weise ich mich besser motivieren kann		… I thought about how I can better motivate myself
**Emotion metalevel**					
Monitoring	EmM1	Diese Woche…	… habe ich über meine Gefühle beim Lernen und Üben nachgedacht	This week…	… I reflected on my feelings while studying and practicing
	EmM2		… habe ich überlegt, welche Gefühle beim Lernen und Üben aufkommen könnten		… I thought about what feelings could arise while studying and practicing
	EmM3		… war ich mir meiner Gefühle beim Lernen und Üben bewusst		… I was aware of my feelings while studying and practicing
	EmM4		… habe ich auf meine Gefühle beim Lernen und Üben geachtet		… I paid attention to my feelings while studying and practicing
	EmM5		… habe ich überlegt, ob mich meine Gefühle beim Lernen und Üben beeinträchtigen		… I thought about whether my feelings are impeding me while studying and practicing
Control	EmC1	Diese Woche…	… habe ich etwas geändert, wenn ich gemerkt habe, dass mich meine Gefühle (z.B. Angst oder Ärger) beim Lernen oder Üben beeinträchtigen	This week…	… I changed something when I noticed that my feelings (e.g., fear or anger) were impeding me while studying or practicing
	EmC2		… habe ich überlegt, wie ich mit meinen Gefühlen beim Lernen und Üben umgehen werde		… I thought about how to deal with my feelings while studying and practicing
	EmC3		… konnte ich mit meinen Gefühlen beim Lernen und Üben gut umgehen		… I was able to deal well with my feelings while studying and practicing
	EmC4		… kam ich mit emotional herausfordernden Situationen gut zurecht		… I coped well with emotionally challenging situations
	EmC5		… habe ich überlegt, auf welche Art und Weise ich besser mit meinen Gefühlen beim Lernen und Üben umgehen kann		… I thought about how I can better deal with my feelings while studying and practicing
**Context metalevel**					
Monitoring	CnM1	Diese Woche…	… habe ich darüber nachgedacht, welche Rahmenbedingungen^1^ mein Lernen und Üben begleiten	This week…	… I reflected on what contextual conditions^1^ accompany my studying and practicing
	CnM2		… habe ich überlegt, welche Rahmenbedingungen^1^ auf mich zukommen werden		… I thought about what contextual conditions^1^ I will encounter
	CnM3		… habe ich über die Rahmenbedingungen^1^ nachgedacht		… I reflected on contextual conditions^1^
	CnM4		… habe ich auf die Rahmenbedingungen^1^ geachtet		… I paid attention to contextual conditions^1^
	CnM5		… habe ich mir die Rahmenbedingungen^1^ für mein Lernen und Üben bewusst gemacht		… I made myself aware of the contextual conditions^1^ of my studying and practicing
Control	CnC1	Diese Woche…	… habe ich die Art und Weise, wie ich lerne oder übe, geändert, um mich an die Rahmenbedingungen^1^ besser anzupassen	This week…	… I changed how I study or practice in order to better adapt to contextual conditions^1^
	CnC2		… habe ich überlegt, was ich tun kann, um mit ungünstigen Rahmenbedingungen^1^ besser zurecht zu kommen		… I thought about what I can do to better deal with unfavourable contextual conditions^1^
	CnC3		… konnte ich mit ungünstigen Rahmenbedingungen^1^ gut umgehen		… I was able to handle unfavourable contextual conditions^1^ well
	CnC4		… habe ich überlegt, wie ich künftig mit ungünstigen Rahmenbedingungen^1^ vor Ort umgehen werde		… I thought about how I will deal with unfavourable contextual conditions^1^ on-site in the future
	CnC5		… habe ich überlegt, auf welche Art und Weise ich besser mit ungünstigen Rahmenbedingungen^1^ umgehen kann		… I thought about how I can better deal with unfavourable contextual conditions^1^
			^1^(organisatorische Rahmenbedingungen, Lehrende, Mitstudierende, Team vor Ort, Gleichbehandlung)		1(organisational conditions, instructors, other students, on-site staff, equity concerns)

All scales were administered using a five-point Likert scale with 1 = *does not apply at all*, 2 = *does not apply*, 3 = *partly applies*, 4 = *applies*, 5 = *fully applies;* for the ‘control’ scales at the metalevel, *6 = This case did not occur* was also included; except for the component emotion with 1 = *not at all*; 2 = *a little*; 3 = *moderately*; 4 = *fairly*; 5 = very much. *Reverse coded. The English translation is a simple translation for the manuscript but not a back-and-forth translation by two different persons as is recommended for translating questionnaires

Cognitive learning strategies for workplace learning are different from those for academic learning (Klingsieck, 2018; Pintrich et al., 1993; Weinstein et al., 2010): First, students use cognitive learning strategies not only in the performance phase but also in the preparation and reflection phases. On closer inspection, learning strategies before and after students’ presence in the workplace can be further differentiated (e.g. into rehearsal, elaboration and organization). We decided against further differentiation because it seems more important to measure whether students prepare and consolidate and less how they do this exactly.

Second, proximal metacognitive learning strategies are a newly introduced set of scales specific to workplace learning. In Step 2 and Step 6 the students reported that they learned by planning, reviewing and reflecting on concrete professional medical activities (e.g., monitoring whether they were following the correct medical procedure to take a blood sample) and that these strategies were more important to them than planning, monitoring or reflecting on the learning process at the cognition metalevel (e.g., monitoring the cognitive learning strategies they used to achieve a learning goal).

Whereas the assessment of the nomological network revealed plausible associations between the newly developed cognitive learning strategies scales and established scales, no association between the newly developed proximal metacognitive learning strategies and the established scales were found. An exception was the newly developed ‘reviewing’ scale, which correlated positively with the established ‘regulation’ scale (Klingsieck, 2018). These findings suggest that proximal cognitive learning strategies can be seen as a distinct category of learning strategies specific to the workplace setting, but further research on the nomological network is recommended.

The development of the indicators and scales for the component cognition was a nonlinear process due to divergent feedback from researchers and students. Their views differed not so much in terms of the wording of the items but in terms of the structure of the indicators. Therefore, the list of indicators changed with each step. It is hoped that the inventory now provides a useful set of scales covering the whole cognitive learning process of a student for one day, before, during and after his or her presence in the clinical practice setting. However, the discrepancies in feedback from the researchers and students suggest the need for further research from an educational psychology perspective on learning strategies for the workplace setting.

#### Motivation

The motivation component refers to the initiation and maintenance of goal-directed activity. It consists of seven scales representing stakeholders’ perspectives on relevant motivational aspects of workplace learning (see Table 5). Psychometric analysis revealed the unidimensionality and acceptable reliability of the scales.

The expectancy-value theory (Eccles & Wigfield, 2020), was shown to be relevant not only to academic learning (Pintrich et al., 1993), but also to workplace learning (‘expectancy of success’ and ‘situational interest’). The results of the nomological network assessment were as expected (Kunter et al., 2002).

Also, achievement goal theory (Urdan & Kaplan, 2020) is relevant to both academic and workplace settings. The ‘performance goal approach’ scale in workplace learning needs careful interpretation because it was positively related not only to the ‘performance goal approach’ scale but also to the ‘performance goal avoidance’ scale in academic learning (Schwarzer & Jerusalem, 1999). The scales representing the avoidance component were deleted in the Workplace Learning Inventory due to the risk of biased responses and the already long list of motivational indicators. However, in Step 2 some interviewees reported that avoiding failure when performing medical activities in front of others was also a relevant motivational aspect. Further research is needed to explore achievement goal theory in the context of workplace learning, especially since achievement goal theory has been further developed in recent years (Urdan & Kaplan, 2020).

Effort and attention control have been added to the abovementioned motivational aspects based on expert review and cognitive pretesting. The nomological network analysis showed results as expected. (Boerner et al., 2005). The ‘proactive attitude’ scale addresses a new motivational aspect specific to workplace learning (if someone is willing to take action). Contrary to expectations, ‘proactive attitude’ was not associated with any of the motivational aspects of academic learning. ‘Proactive attitude’ seems to be a distinct indicator in the workplace setting, and further research on the nomological network is needed.

#### Emotion

The emotion component is defined ‘within the broader concept of affect, but differs from other affective phenomena, such as mood, in that emotions are more intense, have a clearer object-focus, a more salient cause, and are typically experienced for a shorter duration’ (Duffy et al., 2018). The emotion component comprises two scales, ‘positive emotions’ and ‘negative emotions. Psychometric analysis showed the unidimensionality and acceptable reliability of the scales. We did not assess the nomological network, as the scales are short versions of the established MES scales.

In the interpretation of emotions in workplace learning, it is important to remember that the terms ‘positive’ and ‘negative’ describe the quality of single emotions but not their effect on achievement. Both positive and negative emotions can help or hinder a learning process. For example, the positive emotion of curiosity can be a motivator, but high levels of curiosity can also lead to getting lost in details. A high level of the negative emotion of frustration can be demotivating, but a low level of frustration can be a motivator to do better next time and lead to higher achievement.

#### Context

The context component focuses on concrete contextual aspects that are relevant, i.e., helpful or detrimental, to undergraduate workplace learning. In our newly developed questionnaire, the context component is represented by the ‘organizational framework conditions’, ‘supervisory quality’, ‘staff support’, ‘peer support’ and ‘equal treatment’ scales. Psychometric analysis revealed the unidimensionality and acceptable reliability of the scales.

The relationships in the nomological network were as expected, with two exceptions: We did not expected the newly developed ‘peer support’ to be associated with the established ‘perception of teacher’ scale. A possible explanation might be that the teacher shapes the learning environment (e.g., classroom structure; Ames, 1992; Bergsmann et al., 2013) and class climate (Allodi, 2010). Additionally, ‘equal treatment’ was not associated with the established scales and further research on the nomological network of this scale is needed.

The newly developed context scales differ from established scales in that they are distinct from the scales addressing cognition, motivation, and emotion at both levels, i.e., the scale and item levels. This is important to avoid construct contamination. Some established learning environment questionnaires use a holistic definition of the learning environment and include cognitive, motivational or emotional aspects of the learning environment (AlHaqwi et al., 2014; Roff, 2005).

Furthermore, the interviews in Step 2 of Study 1 revealed the important role of peers and staff alongside other factors such as supervisory quality, organizational framework conditions, and equal treatment: Students learn not only from the teacher/supervisor but also from peers and other health professionals at the workplace. This is in line with studies on coregulation in SRL (Bransen et al., 2020) and community of practice (Cruess et al., 2018). Peers and staff also address the need for social relatedness. Social relatedness is an important determinant of personal growth according to self-determination theory (Deci & Ryan, 2012). Feeling accepted and supported by people in the workplace is relevant to students and their learning. Therefore, we decided not to integrate peers and staff into a more general ‘atmosphere’ scale or ‘framework conditions’ scale but to provide separate scales.

#### Metalevel

The cognition metalevel, motivation metalevel, emotion metalevel and context metalevel components regulate the respective aspects of the learning process. At this level, students are no longer at the learning process level and instead reflect on their learning process from a meta-perspective. For each of the four components, we included the ‘monitoring’ and ‘control’ scales. The psychometric analysis revealed the unidimensionality and acceptable reliability of the scales. The results regarding the different components on the metalevel are discussed together, as they have some similarities due to equivalent scales.

The inclusion of only two scales for the metalevel components contrasts with the theoretical perspective in educational psychology research on the academic setting, especially for the metalevel of cognition. Metacognition is a well-established and well-researched concept (e.g., see the various questionnaires or scales for the academic setting; Boerner et al., 2005; Edwards et al., 2014; Klingsieck, 2018; Pintrich et al., 1993) that encompasses different aspects. Pintrich (2004), for example, distinguishes among anticipation, planning, monitoring, control, evaluation, and reaction for each area. Contrary to the theoretical perspective, the students reported in the interviews in Step 2 and the cognitive pretesting in Step 6 of Study 1 that they did not think about their learning strategies, motivation, emotion and context in such a differentiated way, although they reported that thinking about their own learning behavior was crucial.

Furthermore, for the cognition metalevel, the cognitive pretesting of the cognition metalevel items revealed that the students thought about regulating concrete medical activities instead of regulating their learning behavior. These results can be interpreted in the context of the discussion about conscious and unconscious self-regulation of learning (Wirth et al., 2020). It is assumed that students regulate their learning unconsciously (i.e., anticipate, plan, monitor, control, evaluate and react) except in situations where they are faced with difficulties or challenging tasks (Flavell, 1979; Wirth et al., 2020). The decision to use equivalent scales for each component on the metalevel is supported by the findings of Kim and colleagues (Kim et al., 2020), who found that the cognition, motivation and emotion metalevels share regulatory mechanisms. Further research is needed to investigate whether the cognition, motivation, emotion, and context metalevels in workplace learning also share regulatory mechanisms.

The results of the nomological network for the metalevel components are complex. In the interpretation of the nomological network for the motivation and emotion metalevels, the different measurement foci must be taken into account. The newly developed scales focus on the question of *whether* students regulate motivation, emotion and the perception of context in contrast to established scales focusing on the *how*. We first highlight the most important results for the respective ‘monitoring’ scale and then for the ‘control’ scale.

For the cognition metalevel, the results are as expected. For the motivation metalevel, the newly developed ‘monitoring’ scale was positively associated with established scales (Schwinger et al., 2007) that are more relevant to the current situation and time e.g., increasing situational interest (Schraw & Lehman, 2001), but not with strategies that are relevant at a later time, e.g., a good grade. For the emotion metalevel, the newly developed ‘monitoring’ scale was positively associated with the established ‘rumination’ and ‘catastrophization’ scales but also with the ‘refocusing on planning’ scale (Garnefski et al., 2001; Loch et al., 2011). The association with rumination is consistent with theoretical considerations, as rumination refers to thinking about emotions (Loch et al., 2011), although monitoring does not necessarily involve rumination in the sense of becoming stuck. The interpretation of the association with catastrophizing and refocusing on planning is more complex. This could indicate that students who monitor their emotions use detrimental strategies to deal with negative emotions in addition to the helpful strategy of refocusing on planning. It could also indicate a process of dealing with emotions that begins with detrimental strategies such as rumination and catastrophizing before refocusing on planning. For context metalevel established questionnaires were missing.

Regarding the ‘control’ scales, the assessment of the nomological network showed no association with the established scales. A possible explanation for this result is the different level of scale-specificity. While the newly developed scales are on a more general level, the established scales are on a more specific level.

### Strengths and limitations

To ensure the identification of indicators relevant to workplace learning and to address different types of validity, we combined qualitative and quantitative methods and included participants with different perspectives according to Gehlbach and Brinkworth’s seven steps (2011). To enhance trustworthiness, the questionnaire was developed by a multidisciplinary team that included members with different perspectives (Patton, 1999).

Our study also has some limitations because each component is a separate field of research and could be studied separately and in more depth. For example, for the area of context, the interpersonal aspects of learning between the learner and the faculty are less emphasized in the Workplace Learning Inventory (Cruess et al., 2018; Deci & Ryan, 2012; Morris & Behrens, 2013a; Roff & McAleer, 2017). Furthermore, the relationship of the newly developed scales within their nomological networks needs further attention in future studies. In the absence of established questionnaires for assessing workplace learning, we used established questionnaires for the academic setting. While it can be assumed that there is a relationship between the learning components of the academic setting and the workplace setting, this needs further investigation. Another limitation of our study is that the participants for the psychometric analysis came from only two institutions, both targeting the same health profession. We assume that the questionnaire is appropriate for different health professions because (a) the items are not specific to one health profession or field; (b) the students were in heterogeneous workplace settings; and (c) the scales and items were developed by integrating the perspectives from students, teachers and researchers from different institutions and health professions. However, results should be validated using samples from other health professions to find out, whether the items measure the same in related disciplines.

### Scientific and practical implications

With regard to scientific implications, we highlight three needs that our study addresses. They have been articulated by the scientific community in relation to health sciences education. First, Albert et al. (2020) showed the need for interdisciplinarity in research on health sciences education. We address this need by integrating the educational psychology perspective on workplace learning. This is also in line with the tradition regarding research on workplace learning, where interdisciplinarity is highly valued (Hager, 2013). Second, van Houten-Schat et al. (2018) indicated the need to “unravel the sub-processes of SRL that are relevant to the clinical context in order to contribute to more elaborate SRL frameworks for this specific context” (p. 1014). They also determined the need for more quantitative studies. We address these needs by providing scales for the eight most relevant components of SRL in the workplace context which researcher can then select from based on the specific SRL model and research question. This is also in line with the call for a more holistic perspective in educational psychology research connecting different components of learning (Pekrun, 2006; Richardson et al., 2012). Third, researchers (Ciere et al., 2015; Schmitz & Perels, 2011; Schmitz et al., 2011) highlighted the potential of quantitative diary methods in studying learning in the healthcare setting. We address this issue by providing short scales and by formulating items that, viewed on a trait-state continuum (Geiser et al., 2017), address the state aspect of learning more.

With regard to practical implications, a better understanding of workplace learning can help address several problems in the practical part of health sciences education, two of which we highlight. First, a better understanding of the transition from academic learning to workplace learning addresses the problem of students struggling during the transition period (Atherley et al., 2019; Godefrooij et al., 2010; Teo et al., 2011). Students often perceive transition situations in health sciences education to be challenging and stressful (Teunissen & Westerman, 2011; Westerman & Teunissen, 2013). A better understanding of workplace learning can serve as a basis for intervention or further improvement of the curriculum. Second, a better understanding of workplace learning can address the problem of low well-being among health science students and professionals. This is especially important, as distress, depression and anxiety are severe issues (Dyrbye et al., 2006; Hope & Henderson, 2014). A better understanding of students’ workplace learning can help to identify unfavorable trends not only in student achievement but also in students’ well-being and serve as a basis for developing preventive measures.

### Conclusion

The newly developed Workplace Learning Inventory is the first to address undergraduates’ workplace learning from an educational psychology research perspective. It is very comprehensive, as it addresses four different areas at two different levels, resulting in eight components of learning. Each component is addressed by several indicators and scales. The newly developed scales are short so that their administration is feasible in the workplace setting and they do not overlap and can therefore be combined in multivariate studies.

By providing the Workplace Learning Inventory, we hope to encourage multivariate studies of undergraduate workplace learning. Future studies can use the inventory for comprehensive investigations of undergraduate workplace learning in a cross-sectional or short-term longitudinal study by implementing a broad range of scales and for more detailed investigations of specific aspects in long-term longitudinal studies by selecting the respective scales. Such studies could contribute to a better understanding of workplace learning, its development over time and the associations between SRL components and other concepts relevant to workplace learning, such as stress or empathy.

### Supplementary Information

Below is the link to the electronic supplementary material.

Supplementary file1 (PDF 384 kb)

## Footnotes

1 Both, Boekaerts and Wirth and colleagues propose a third level, the domain-specific knowledge level or the content layer, respectively. We propose a model for workplace learning assessment specific to health sciences education, but not specific to a domain such as human medicine, veterinary medicine, nursing, physiotherapy or similar domains. As we are proposing a more general model for workplace learning assessment in health sciences education, we exclude this domain-specific layer from our model.
