# Supplementary material for: Development and validation of the Workplace Learning Inventory in Health Sciences Education: a multimethod study
Source: Adv Health Sci Educ Theory Pract. 2023 Nov 8;29(4):1075–129. doi: 10.1007/s10459-023-10295-y (PMC11369049; doi:10.1007/s10459-023-10295-y)
Supplement: Supplementary file 1 — Supplementary file1 (PDF 384 kb) [file 10459_2023_10295_MOESM1_ESM.pdf]

Supplement material

Development and validation of the Workplace Learning Inventory in Health Sciences Education: a multimethod study

Evelyn Steinberg · Stephan Marsch · Takuya Yanagida · Laura Dörrenbächer-Ulrich · Christopher Pfeiffer · Petra Bührle · Lukas Schwarz · Ulrike Auer · Christin Kleinsorgen · Franziska Perels

In the following, we make the process of Study 1 transparent by describing the development of the indicators and items of each component. Table 1 shows the list of indicators after each step for each of the eight components. (Table 1 is included at the end of this document.) The process of developing the indicators was not linear, but the indicators were discussed and revised throughout the development process.

Cognition

For cognition, Table 1 shows a discrepancy between the indicators derived from established questionnaires (Step 1) and those derived from the interviews (Step 2). The interviewees' statements on workplace learning revealed a process-oriented perspective on learning strategies with a focus on learning from the regulation of professional medical activities: undergraduates - as well as medical professionals - need to anticipate, plan and prepare for upcoming professional medical activities; they need to review whether they are doing a medical procedure correctly and adjust their activity if necessary; and afterward, they need to evaluate what was done well and what needs to be done differently next time, and they need to react by implementing their insights next time. The following statement from a student illustrates this perspective on anticipation, planning and preparation:

> For example, in the case of major surgeries that I have never seen before, I take a quick look at the most important key facts in the evening or sometimes in the morning. [...] I now also take a look at the surgery schedule. What is the daily schedule? Where do I have to go? (Student 3)

Due to discrepancies in the categorization of indicators between those derived from Step 1 and those derived from Step 2, the project team decided to include only those derived from interviewees' statements in the synthesized list, because it prioritized the stakeholder perspective over the indicators derived from the academic learning literature (Step 3). We developed items corresponding to the selected indicators (Step 4) and revised them based on the expert review (Step 5). However, at this point in the process, when reviewing the revised items, the project team concluded that the list of items related to cognition did not cover all aspects of the cognitive component. We

added the indicators attention, rehearsal, elaboration, and organization, which are included in the MSLQ (Pintrich et al., 1993). In doing so, we reformulated the established items for academic learning from the German questionnaire Learning Strategies of University Students (LIST; Klingsieck, 2018; Wild & Schiefele, 1994), because it is based on the MSLQ, to address workplace learning. As these indicators address the knowledge part of workplace learning, we added the newly developed indicator 'learning by doing' to also address the skills aspect of workplace learning. Because of their close connection and to reduce the number of scales, we combined the newly developed scales 'anticipation' and 'planning' into 'preparation' and combined 'evaluation' and 'reaction' into 'consolidation'; in addition, we renamed 'monitoring' and 'control' as 'reviewing' and 'clarification' to distinguish these indicators from those for the cognition metalevel.

Based on the results of the cognitive pretest (Step 6), we dropped the learning by doing items because they were confounded by a fairness aspect: Students often want to take action, but to be fair, they often let a peer go first. This was illustrated by one student's statement:

> If I feel confident, I let others go first; there are plenty of opportunities to practice. (Student 2)

After cognitive pretesting, we decided to introduce two subcomponents for cognition to clarify the difference between 'traditional' cognitive strategies and newly developed strategies for workplace learning. (1) Cognitive learning strategies include preparation, attention, rehearsal, elaboration, clarification and consolidation (similar to the MSLQ, Pintrich et al., 1993; and LASSI (Edwards et al., 2014). (2) Proximal metacognitive learning strategies are strategies in which students learn from regulating professional medical activities, including planning, reviewing, and reflection; students do not regulate their learning process (metacognition, Boekaerts, 1997; Wirth & Leutner, 2008) but their professional medical activities.

Motivation

For motivation, Table 1 shows a correspondence between indicators from established questionnaires (Step 1) and indicators reported by the stakeholders (Step 2). In the synthesized list (Step 3), we decided to include indicators representing two theories, which are considered as main theories in recent motivation research (Koenka, 2020), namely, expectancy-value theory (Eccles & Wigfield, 2020) and achievement goal theory (Urdan & Kaplan, 2020). The existing items were rephrased to reflect the workplace setting (Step 4).

Expectancy-value theory is represented by 'expectancy of success' and 'interest'. 'Interest' was further divided into 'situational interest' and 'individual interest' (Hidi & Renninger, 2006). The interviewees (Step 2) reported that individual interest is very important in workplace learning:

> It is positive if I find the field of the workplace interesting. [...] It’s certainly negative if it’s a discipline where I know I don’t want to do it anyway, it’s of no use to me. (Student 1)

> Interest in the field, interest in the profession, that you can experience the profession. Then, there is specific interest in the subject. That can be very inhibiting; if someone wants to be a surgeon and comes into psychiatry, they may not have any interest at all in dealing with schizophrenic patients. (Teacher 6)

However, we dropped the ‘individual interest’ indicator as a result of the cognitive pretesting (Step 6). The students reported that the more general wording of the items was too vague:

> What are ‘career goals’? I do not know what this means, especially when you do not yet know where you want to go. (Student 2)

The students recommended instead using a more concrete list of medical fields to determine more about individual interests. As such a list was specific to the target group, we decided to not include ‘individual interest’.

Achievement goal theory was represented in the synthesized list by the mastery and performance approach indicators and their avoidance indicators (Step 3). The existing items were reformulated to reflect the workplace context (Step 4). In the cognitive pretest (Step 6), some students reported that the mastery goal avoidance items as well as the performance goal avoidance items were at risk of not being answered honestly due to social desirability:

> You might be afraid of being perceived as arrogant if you report wanting to do better than others. (Student 6)

Due to the risk of biased responses and the already long list of motivational indicators, we removed the avoidance indicators. Although attributions were mentioned by interviewees (Step 2), we did not include this concept in the synthesized list (Step 3) because according to attribution theory, at least six more indicators would have had to be included, which we considered to be overwhelming due to an already long list of indicators.

In addition to the two main theories, three indicators were identified as relevant to workplace learning in Steps 5 and 6. The experts (Step 5) recommended the inclusion of effort and attention control as a relevant motivational concept; for example, one expert noted:

> For me, this item [representing attention] also has a strong motivational/volitional aspect in the sense of not letting yourself to be distracted. (Expert 3)

During the cognitive pretesting (Step 6), some students pointed out that the cognition indicator 'learning by doing' was less relevant than the will to take action. Therefore, the indicator 'proactive attitude' was added based on reformulated items of the former 'learning by doing' indicator.

Emotion

For emotion, Table 1 shows that the categorization of emotions into positive and negative emotions based on the literature (Step 1) and in particular on the MES (Duffy et al., 2018) as well as on the interviews (Step 2) was useful. While the literature also includes neutral emotions (e.g., neutral), we removed this indicator from the synthesized list (Step 3), as it was not important to the interviewees and was represented by only one emotion in the literature. The interviewees' statements (Step 2) did not reveal any additional emotions beyond those listed in the MES. Therefore, we decided to rely on the items of the MES. As the MES is an established questionnaire for workplace learning in medical education, expert review and cognitive pretesting were redundant.

Context

For context, Table 1 shows the indicators from two questionnaires (Step 1). The categorization of the scales differs between these two questionnaires, and therefore, the scales are not distinct from each other. Additionally, several items of established context scales did not fit our definition of context (e.g., motivation of the CLEQ, AlHaqwi et al., 2014). In analyzing the interviewees' statements (Step 2), we categorized the statements within the UCEEM (Strand et al., 2013) indicators since they fit best and excluded aspects from other areas (e.g., motivation). However, we identified several subcategories. For the synthesized list (Step 3), we decided to further develop the UCEEM indicators based on the interviewees' statements: We differentiated between actors that are part of the learning environment and could coregulate (Bransen et al., 2020) students in their learning process, namely, teachers/supervisors, peers and staff (clinical team). The provision of physical resources and preparedness for student entry were combined into the indicator 'organizational preparedness'. The indicator 'equal treatment' was retained. The formulation of the items is based on the interviewees' statements (Step 2) and on the UCEEM. No changes were made to the indicators during the expert review (Step 5) and the cognitive pretest (Step 6), but some items were deleted or rephrased. An exception was the renaming of 'organizational preparedness' to 'organizational framework conditions'.

Cognition Metalevel

For the cognition metalevel, Table 1 shows an overlap between indicators derived from established questionnaires (Step 1) and indicators derived from the interviews (Step 2). In the interviews, we asked the interviewees in general terms about useful strategies at the cognition metalevel and then, more specifically, whether Pintrich's (2004) categorization of anticipation, planning, monitoring, control, evaluation, and reaction made sense to them. The answers were heterogeneous:

> Most of the time, you have the feeling that while they [the students] are in the clinic, I think, they are much too busy to think about it [their learning] again but that they usually only come up with it when you discuss it with them. So at least I have the feeling that it's not while being in the clinic because there's way too much going on. (Teacher 1)

> If you want to be able to regulate emotion, motivation, cognition, and perception of the learning environment, you should also think about the metalevel of that aspect. (Researcher 2)

We maintained the differentiation of these aspects due to the strong support in the literature (Step 3) and developed items accordingly (Step 4). While no changes were made after the expert review (Step 5), during the cognitive pretesting (Step 6), the students reported thinking more about concrete medical activities (thinking about medical activities) and less about their learning process (thinking about their learning behavior) when answering the items. They also reported thinking less about their learning process:

> I don't think that much about my own learning; this was difficult to answer. I can answer these items at the end of the course rather than at the end of the day. (Student 5)

Based on the students' feedback and on the suggestion of Wirth and colleagues, we reduced the indicators to 'monitoring' and 'control' only.

Motivation Metalevel

For the motivation metalevel, Table 1 shows an established questionnaire on effort regulation in academic learning, which includes different strategies to increase motivation (Step 1). In the interviews (Step 2), we did not ask how the students regulated motivation but whether they thought about motivation on a metalevel. The interviewees reported that monitoring/evaluation as well as control/reaction were relevant. As the interviewees reported that they did not think about the metalevel in such a differentiated way (see statements on cognition metalevel), we decided to combine the different aspects into a single indicator 'regulation of motivation' similar to the indicator of the MLSQ (Pintrich et al., 1993) (Step 3) and developed corresponding items (Step 4). However, after the

cognitive pretesting, we decided to split the indicator into ‘monitoring’ and ‘control’ (Nelson & Narens, 1990) to be consistent with the metalevel of cognition.

Emotion Metalevel

For the emotion metalevel, Table 1 shows established questionnaires on emotion regulation, which are the AERQ and CERQ (Step 1). These questionnaires are not specific to the learning context but ask more generally about different strategies for dealing with negative emotions. The results for the emotion metalevel from the interviews (Step 2) and the further development of the indicators run parallel to the indicators for the motivation metalevel.

Context Metalevel

For the context metalevel, we found no established scales that are applicable or transferable to workplace learning (Step 1). The results for the context metalevel from the interviews (Step 2) and the further development of the indicators run parallel to the indicators for the motivation metalevel.

Table 1

List of Indicators per Component and Step

Component	Step 1<br><br>Indicators derived from literature	Step 2<br><br>Indicators derived from interviews	Step 3<br><br>Synthesized list of indicators	Step 5<br><br>Indicators after expert review	Step 6<br><br>Indicators after cognitive pretesting
Cognition	MSLQ Cognitive strategies<br>• Rehearsal<br>• Elaboration<br>• Organisation<br>• Critical thinking<br>LASSI Information processing scale<br>• Imagery<br>• Verbal elaboration<br>• Organization strategies<br>• Reasoning skills<br>• Selecting main ideas scale	• Anticipation<br>• Planning<br>• Monitoring<br>• Control<br>• Evaluation<br>• Reaction<br><br>(regarding professional medical activities)	• Anticipation<br>• Planning<br>• Monitoring<br>• Control<br>• Evaluation<br>• Reaction<br><br>(regarding professional medical activities)	• Preparation<br>• Attention<br>• Rehearsal<br>• Elaboration<br>• Organisation<br>• Learning by doing<br>• Reviewing<br>• Clarification<br>• Consolidation	Cognitive learning strategies<br>• Preparation<br>• Attention<br>• Rehearsal<br>• Elaboration<br>• Clarification<br>• Consolidation<br>Proximal metacognitive learning strategies<br>• Planning<br>• Reviewing<br>• Reflection

Motivation	MSLQ Motivation scales<br>• Intrinsic goal orientation<br>• Extrinsic goal orientation<br>• Task value<br>• Control beliefs<br>• Self-efficacy for learning and performance<br>LASSI<br>• Attitude scale<br>• Motivation scale	• Expectation of success and related constructs (self-efficacy, self-worth, self-confidence)<br>• Interest (especially individual interest but also situational interest)<br>• Achievement goals (especially task-approach, self-approach and other-avoidance)<br>• Attributions (especially internal attribution of experiences of success, having controllable attributional beliefs)	• Expectancy of success<br>• Individual interest<br>• Situational interest<br>• Mastery approach<br>• Mastery avoidance<br>• Performance approach<br>• Performance avoidance	• Expectancy of success<br>• Individual interest<br>• Situational interest<br>• Mastery approach<br>• Mastery avoidance<br>• Performance approach<br>• Performance avoidance<br>• Attention control<br>• Effort	• Expectancy of success<br>• Situational interest<br>• Mastery approach<br>• Performance approach<br>• Effort<br>• Attention control<br>• Proactive attitude
Emotion	MES<br>• Negative emotions	• Negative emotions<br>• Positive emotions	• Negative emotions<br>• Positive emotions	• Negative emotions<br>• Positive emotions	• Negative emotions<br>• Positive emotions

	• positive emotions<br>• neutral emotions				
Context	UCEEM<br>• Opportunities to learn in and through work & quality of supervision<br>• Preparedness for student entry<br>• Workplace interaction patterns & inclusion of students<br>• Equal treatment<br>CLEQ<br>• Cases<br>• Authenticity of clinical experiences<br>• Supervision<br>• Organization of the doctor patient encounter	• Opportunities to learn in and through work & quality of supervision<br>– supervisors' teaching professionalism<br>– supervisors' clinical professionalism<br>– supervisors' teaching strategies for content learning<br>– supervisors' teaching strategies for co-regulation of the learning process<br>– a learning-oriented peer group	• Supervisory quality<br>• Organisational preparedness<br>• Staff support<br>• Peer support<br>• Equal treatment	• Supervisory quality<br>• Organisational preparedness<br>• Staff support<br>• Peer support<br>• Equal treatment	• Organisational framework conditions<br>• Supervisory quality<br>• Staff support<br>• Peer support<br>• Equal treatment

	• Motivation/learning skills	• Preparedness for student entry<br>− institutions that put value on student learning<br>• Workplace interaction patterns & inclusion of students<br>− provision of physical resources<br>− social relatedness and learning from peers<br>− social relatedness and learning from clinical team<br>• Equal treatment

Cognition metalevel	MSLQ<br>• Metacognitive self-regulation<br>LASSI<br>• Test strategy scale<br>• Self-regulation component of strategic learning scale<br>• Concentration<br>• Self testing<br>• Time management<br>• Using academic resources	• Anticipation<br>• Planning<br>• Monitoring<br>• Control<br>• Evaluation<br>• Reaction<br>(regarding cognitive aspects of the learning process)	• Anticipation<br>• Planning<br>• Monitoring<br>• Control<br>• Evaluation<br>• Reaction<br>(regarding the cognitive learning process)	• Anticipation<br>• Planning<br>• Monitoring<br>• Control<br>• Evaluation<br>• Reaction<br>(regarding the cognitive learning process)	• Monitoring<br>• Control<br>(regarding the cognitive learning process)
Motivation metalevel	EFFORT REGULATION<br>• Increasing situational interest<br>• Increasing personal value	• Monitoring/Evaluation<br>• Control/Reaction<br>(regarding motivational aspects of the learning process)	• Regulation of motivation	• Regulation of motivation	• Monitoring<br>• Control<br>(regarding motivational aspects of the learning process)

	• Performance-goal-approach oriented self-instruction<br>• Self-rewarding<br>• Learning-goal-approach oriented self-instruction<br>• Controlling learning environment<br>• Performance-goal-avoidance oriented self-instruction<br>• Setting subgoals				
Emotion metalevel	AERQ<br>• Avoiding situations<br>• Developing competences<br>• Redirecting attention<br>• Reappraisal<br>• Suppression	• Monitoring/Evaluation<br>• Control/Reaction<br><br>(regarding emotional aspects of the learning process)	• Regulation of emotion	• Regulation of emotion	• Monitoring<br>• Control<br><br>(regarding emotional aspects of the learning process)

	• Respiration<br>• Venting<br>• Seeking social support<br>CERQ<br>• Self-blame<br>• Other-blame<br>• Rumination<br>• Catastorphizing<br>• Putting into persepctive<br>• Positive refocusing<br>• Positive reappraisal<br>• Acceptance<br>• Refocus				
Context metalevel	-	• Control/Reaction<br><br>(regarding context aspects of the learning process)	• Regulation of context	• Regulation of context	• Monitoring<br>• Control<br><br>(regarding context aspects of the learning process)

Note: At the learning process level, we considered the two most widely used SRL questionnaires (Roth et al., 2016); where the SRL questionnaires did not provide sufficient information we considered questionnaires on specific indicators. At the metalevel, we considered established SRL questionnaires for metacognition and for regulation of motivation and emotion. Please note that Step 4 is developing items and hence not listed in the table showing indicators for each component. MSLQ = Motivated Strategies for Learning Questionnaire (Pintrich et al., 1993); LASSI = Learning and Study Strategies Inventory (Edwards et al., 2014); MES = Medical Emotion Scale (Duffy et al., 2018); EFFORT REGULATION (Schwinger et al., 2007; Wolters, 1998, 1999); UCEEM = Undergraduate Clinical Environment Education Measure (Strand et al., 2013); CLEQ = Clinical Learning Evaluation Questionnaire (AlHaqwi et al., 2014); AERQ = Emotion Regulation Questionnaire (Burić et al., 2016); CERQ = Cognitive Emotion Regulation Questionnaire (Loch et al., 2011).

References

AlHaqwi, A. I., Kuntze, J., & van der Molen, H. T. (2014). Development of the clinical learning evaluation questionnaire for undergraduate clinical education: Factor structure, validity, and reliability study. *BMC Medical Education*, *14*, 44. https://doi.org/10.1186/1472-6920-14-44

Boekaerts, M. (1997). Self-regulated learning: A new concept embraced by researchers, policy makers, educators, teachers, and students. *Learning and Instruction*, *7*(2), 161–186. https://doi.org/10.1016/S0959-4752(96)00015-1

Bransen, D., Govaerts, M.J.B., Sluijsmans, D. M. A., & Driessen, E. W. (2020). Beyond the self: The role of co-regulation in medical students' self-regulated learning. *Medical Education*, *54*(3), 234–241. https://doi.org/10.1111/medu.14018

Burić, I., Sorić, I., & Penezić, Z. (2016). Emotion regulation in academic domain: Development and validation of the academic emotion regulation questionnaire (AERQ). *Personality and Individual Differences*, *96*, 138–147. https://doi.org/10.1016/j.paid.2016.02.074

Duffy, M. C., Lajoie, S. P., Pekrun, R., & Lachapelle, K. (2018). Emotions in medical education: Examining the validity of the Medical Emotion Scale (MES) across authentic medical learning environments. *Learning and Instruction.* Advance online publication. https://doi.org/10.1016/j.learninstruc.2018.07.001

Eccles, J. S., & Wigfield, A. (2020). From expectancy-value theory to situated expectancy-value theory: A developmental, social cognitive, and sociocultural perspective on motivation. *Contemporary Educational Psychology*, *61*, 101859. https://doi.org/10.1016/j.cedpsych.2020.101859

Edwards, A. J., Weinstein, C., Goetz, E. T., & Alexander, P. A. (2014). *Learning and Study Strategies: Issues in Assessment, Instruction, and Evaluation*. Elsevier Science.

Hidi, S., & Renninger, K. A. (2006). The Four-Phase Model of Interest Development. *Educational Psychologist*, *41*(2), 111–127. https://doi.org/10.1207/s15326985ep4102_4

Klingsieck, K. B. (2018). Kurz und knapp – die Kurzskala des Fragebogens „Lernstrategien im Studium" (LIST) [A short version of the questionnaire on learning strategies in higher education (LIST)]. *Zeitschrift Für Pädagogische Psychologie*, *32*(4), 249–259. https://doi.org/10.1024/1010-0652/a000230

Koenka, A. C. (2020). Academic motivation theories revisited: An interactive dialog between motivation scholars on recent contributions, underexplored issues, and future directions. *Contemporary Educational Psychology*, *61*, 101831. https://doi.org/10.1016/j.cedpsych.2019.101831

Loch, N., Hiller, W., & Witthöft, M. (2011). Der Cognitive Emotion Regulation Questionnaire (CERQ). *Zeitschrift Für Klinische Psychologie Und Psychotherapie*, *40*(2), 94–106. https://doi.org/10.1026/1616-3443/a000079

Nelson, T. O., & Narens, L. (1990). Metamemory: A Theoretical Framework and New Findings. In *Psychology of Learning and Motivation* (Vol. 26, pp. 125–173). Elsevier. https://doi.org/10.1016/S0079-7421(08)60053-5

Pintrich, P. R. (2004). A Conceptual Framework for Assessing Motivation and Self-Regulated Learning in College Students. *Educational Psychology Review*, *16*(4), 385–407. https://doi.org/10.1007/s10648-004-0006-x

Pintrich, P. R., Smith, D. A. F., Garcia, T., & Mckeachie, W. J. (1993). Reliability and Predictive Validity of the Motivated Strategies for Learning Questionnaire (Mslq). *Educational and Psychological Measurement*, *53*(3), 801–813. https://doi.org/10.1177/0013164493053003024

Roth, A., Ogrin, S., & Schmitz, B. (2016). Assessing self-regulated learning in higher education: a systematic literature review of self-report instruments. *Educational Assessment, Evaluation and Accountability*, *28*(3), 225–250. https://doi.org/10.1007/s11092-015-9229-2

Schwinger, M., Laden, T. von der, & Spinath, B. (2007). Strategien zur Motivationsregulation und ihre Erfassung [Strategies for the regulation of motivation and their assessment]. *Zeitschrift Für Entwicklungspsychologie Und Pädagogische Psychologie*, *39*(2), 57–69. https://doi.org/10.1026/0049-8637.39.2.57

Strand, P., Sjöborg, K., Stalmeijer, R., Wichmann-Hansen, G., Jakobsson, U., & Edgren, G. (2013). Development and psychometric evaluation of the Undergraduate Clinical Education Environment Measure (UCEEM). *Medical Teacher*, *35*(12), 1014–1026. https://doi.org/10.3109/0142159X.2013.835389

Urdan, T., & Kaplan, A. (2020). The origins, evolution, and future directions of achievement goal theory. *Contemporary Educational Psychology*, *61*, 101862. https://doi.org/10.1016/j.cedpsych.2020.101862

Wild, K. P., & Schiefele, U. (1994). Lernstrategien im Studium: Ergebnisse zur Faktorenstruktur und Reliabilität eines neuen Fragebogens [Learning strategies of university students: Factor structure and reliability of a new questionnaire]. *Zeitschrift Für Differentielle Und Diagnostische Psychologie*, *15*(4), 185–200. https://psycnet.apa.org/record/1996-85746-001

Wirth, J., & Leutner, D. (2008). Self-Regulated Learning as a Competence. *Zeitschrift Für Psychologie / Journal of Psychology*, *216*(2), 102–110. https://doi.org/10.1027/0044-3409.216.2.102

Wolters, C. A. (1998). Self-regulated learning and college students' regulation of motivation. *Journal of Educational Psychology*, *90*(2), 224–235. https://doi.org/10.1037/0022-0663.90.2.224

Wolters, C. A. (1999). The relation between high school students' motivational regulation and their use of learning strategies, effort, and classroom performance. *Learning and Individual Differences*, *11*(3), 281–299. https://doi.org/10.1016/S1041-6080(99)80004-1
